# Microbial Consortiums of Putative Degraders of Low-Density Polyethylene-Associated Compounds in the Ocean

**DOI:** 10.1128/msystems.01415-21

**Published:** 2022-03-01

**Authors:** Maria Pinto, Zihao Zhao, Katja Klun, Eugen Libowitzky, Gerhard J. Herndl

**Affiliations:** a Department of Functional and Evolutionary Ecology, University of Vienna, Vienna, Austria; b Research Platform ‘Plastics in the Environment and Society,’ University of Vienna, Vienna, Austria; c Marine Biology Station, National Institute of Biology, Piran, Slovenia; d Department of Mineralogy and Crystallography, University of Vienna, Vienna, Austria; e NIOZ, Department of Marine Microbiology and Biogeochemistry, Royal Netherlands Institute for Sea Research, Utrecht University, Den Burg, The Netherlands; University of Technology Sydney

**Keywords:** LDPE, ocean, biodegradation, biofilms, metagenomics

## Abstract

Polyethylene (PE) is one of the most abundant plastics in the ocean. The development of a biofilm on PE in the ocean has been reported, yet whether some of the biofilm-forming organisms can biodegrade this plastic in the environment remains unknown. Via metagenomics analysis, we taxonomically and functionally analyzed three biofilm communities using low-density polyethylene (LDPE) as their sole carbon source for 2 years. Several of the taxa that increased in relative abundance over time were closely related to known degraders of alkane and other hydrocarbons. Alkane degradation has been proposed to be involved in PE degradation, and most of the organisms increasing in relative abundance over time harbored genes encoding proteins essential in alkane degradation, such as the genes *alkB* and CYP153, encoding an alkane monooxygenase and a cytochrome P450 alkane hydroxylase, respectively. Weight loss of PE sheets when incubated with these communities and chemical and electron microscopic analyses provided evidence for alteration of the PE surface over time. Taken together, these results provide evidence for the utilization of LDPE-associated compounds by the prokaryotic communities. This report identifies a group of genes potentially involved in the degradation of the LDPE polymeric structure and/or associated plastic additives in the ocean and describes a phylogenetically diverse community of plastic biofilm-dwelling microbes with the potential for utilizing LDPE-associated compounds as carbon and energy source.

**IMPORTANCE** Low-density polyethylene (LDPE) is one of the most used plastics worldwide, and a large portion of it ends up in the ocean. Very little is known about its fate in the ocean and whether it can be biodegraded by microorganisms. By combining 2-year incubations with metagenomics, respiration measurements, and LDPE surface analysis, we identified bacteria and associated genes and metabolic pathways potentially involved in LDPE biodegradation. After 2 years of incubation, two of the microbial communities exhibited very similar taxonomic compositions mediating changes to the LDPE pieces they were incubated with. We provide evidence that there are plastic-biofilm dwelling bacteria in the ocean that might have the potential to degrade LDPE-associated compounds and that alkane degradation pathways might be involved.

## INTRODUCTION

Over the last decades, plastic production and consequently plastic pollution in the ocean have drastically increased, with 4.8 to 12.7 million tons of plastic estimated to enter the ocean each year (1). Despite the urgent need for understanding the impact of plastics on marine ecosystems and the growing body of literature on this subject, the fate of plastics in the oceans remains largely enigmatic.

Once in the sea, plastics are rapidly colonized by a diverse microbial community (2). Several studies have focused on describing the taxonomic composition of these communities and the factors that might be responsible for their succession and development (3–12). Microorganisms related to hydrocarbon degraders have been found to be relatively more abundant on specific plastic types than on other plastics and nonplastic surfaces (13–15), especially at the early stages of biofilm formation (8). However, the reasons for these differences in the colonization pattern of different plastic types remain enigmatic. The physical and chemical properties of the plastics and the presence of additives might play important roles in the initial colonization of plastics by microbes and in the subsequent microbial biofilm development (5). However, recent evidence also suggests that plastic biofilm communities are mostly shaped by biogeographic factors (16). Despite these uncertainties, however, there is growing evidence that some organisms forming biofilms on plastic in the ocean do have the ability to degrade plastic-associated compounds (17, 18).

Polyethylene (PE) is the most widely used plastic worldwide. In the year 2018, polyethylene resin demand was estimated to make up 36% of the total nonfiber plastic production (19). At sea, together with polypropylene, PE is usually the most abundant plastic at the sea surface (1, 20).

PE is considered to be highly resistant to biodegradation, especially in the absence of previous weathering by, for instance, photodegradation. However, studies have reported weight loss and changes in the physical and chemical structure of PE when incubated with specific microorganisms. For instance, bacteria of the genera *Bacillus* and *Pseudomonas* have been associated with PE degradation both in the marine environment (21–24) and in soil (25–27). Some studies have also suggested the implication of the *alkB* gene and alkane degradation pathways in PE biodegradation (27, 28).

This *alkB* gene encodes an alkane monooxygenase that oxidizes the initial terminal end of *n*-alkanes. Because the main molecular structure of PE polymers is similar to that of alkanes, it has been suggested that the metabolism responsible of PE biodegradation is similar to that of hydrocarbons, specifically alkanes (29). Alkanes are degraded through a succession of oxidation steps leading to beta-oxidation and, consequently, to the entering of acetyl coenzyme A (acetyl-CoA) into the citric acid cycle. The first oxidation step is the initial terminal hydroxylation of the alkane chains, which can be carried out by enzymes belonging to different families, usually depending on the alkane chain length (30). In organisms degrading short-chained alkanes, it is usually carried out by methane monooxygenase. The initial hydroxylation of medium-chained alkanes (C_5_ to C_17_) is mediated by organisms that frequently contain soluble cytochromes P450, typically the cytochrome P450 CYP153 alkane hydroxylase, and integral membrane nonheme iron monooxygenases, typically AlkB. Alcanivorax borkumensis, for example, has genes encoding both types of medium-chained alkane monooxygenases in its genome (31). Organisms capable of hydroxylating long-chained alkanes (>C_18_), such as Alcanivorax dieselolei and Geobacillus thermoleovorans, have been shown to have the hydroxylases AlmA and LadA, respectively (32, 33). Except for the *alkB*-encoded monooxygenase, any other potential enzyme or pathway that might be involved in PE biodegradation is only poorly studied, particularly in the marine environment.

**FIG 1:**
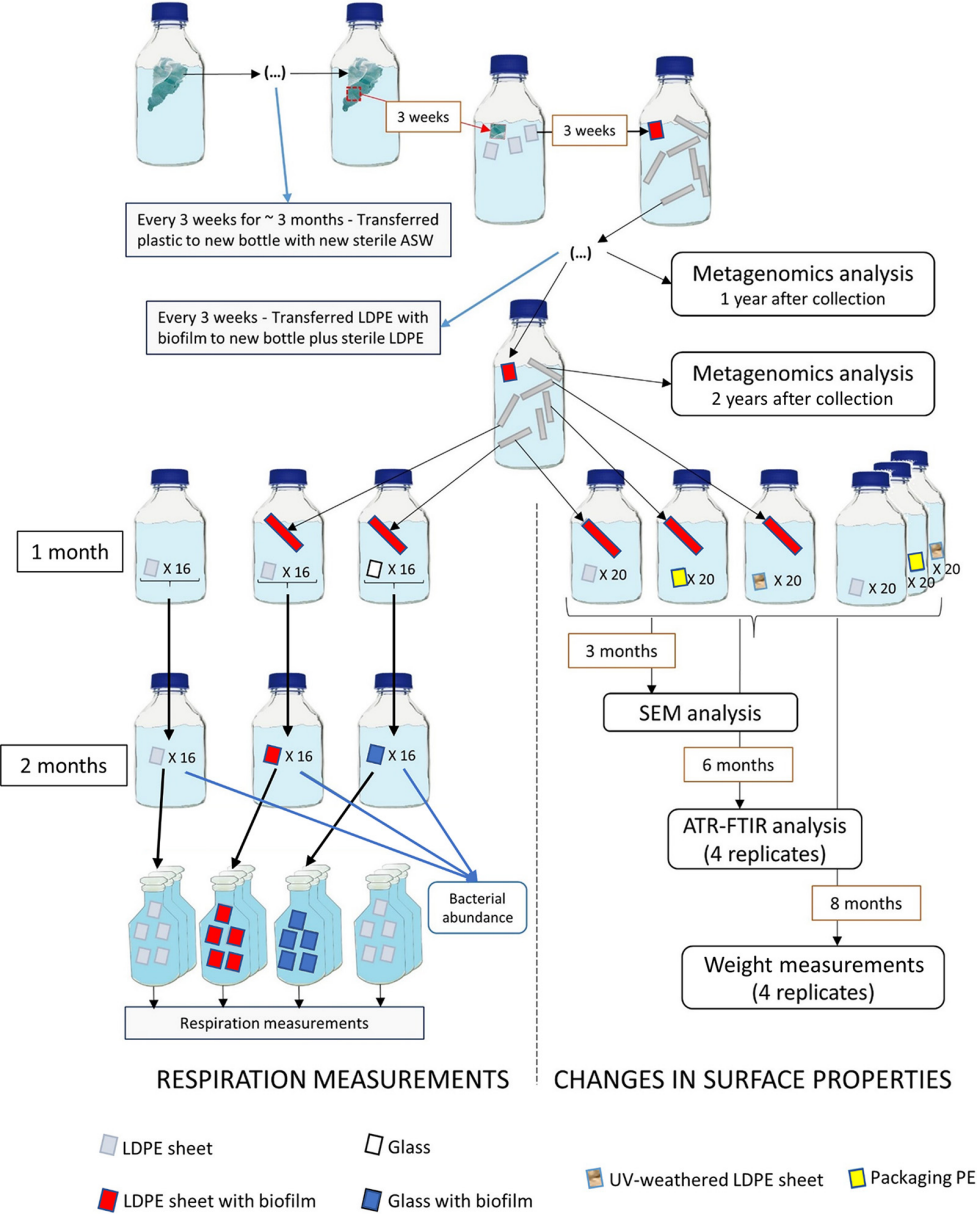
Scheme of the experimental setup. All incubations were performed in sterile artificial seawater amended with 2 μM NaPO_4_ and 10 μM NH_4_Cl. All bottles were kept in the dark at room temperature (∼21°C). This workflow was performed for each of the three plastics, A, B, and C.

In this study, we investigated the potential of three PE biofilm communities (here called communities A, B, and C), initially attached to three different plastics collected from the Northern Adriatic Sea, to metabolize low-density polyethylene (LDPE) and/or associated compounds. The term LDPE-associated compounds used here encompasses the LDPE polymeric chain and any potential additives associated with the used LDPE pieces. The communities were incubated in artificial seawater (ASW) amended with inorganic nutrients and with LDPE as their sole carbon source for 2 years. To identify which taxa and metabolic pathways became enriched during the incubation, metagenomes were analyzed after 1 and 2 years. Oxygen consumption measurements were performed as a metabolic activity parameter to determine whether the microbial communities incubated with LDPE were more active than communities on inert surfaces such as glass. Weighing of the plastic, scanning electron microscopy (SEM), and attenuated total reflection Fourier-transform infrared (ATR-FTIR) spectroscopy were performed on different types of PE sheets to determine whether any physical and/or chemical changes to the surface of the plastic occurred due to the presence of the microbial communities (see Fig. 1 for experimental setup).

## RESULTS

### Taxonomic and functional classification of the microbial communities.

In total, we obtained six metagenomes, one from the original community A (community A0), one for each of the communities after 1 year of incubation (communities A1, B1, and C1) and one for each of the communities A and C after 2 years of incubation (communities A2 and C2, respectively). mOTU (operational taxonomic unit obtained with the mOTUs software) diversity and richness of community A decreased from the original community (A0) to the community after being incubated for 1 year in the case of community A (A1). Diversity and richness also decreased from year 1 to year 2 in communities A and C (A2 and C2) (see Fig. S1 in the supplemental material).

10.1128/msystems.01415-21.3 FIG S1 OTU diversity indices and richness of the six metagenomes calculated from the OTUs. Download FIG S1, TIF file, 1.0 MB. Copyright © 2022 Pinto et al. 2022 Pinto et al. https://creativecommons.org/licenses/by/4.0/ This content is distributed under the terms of the Creative Commons Attribution 4.0 International license.

**FIG 2:**
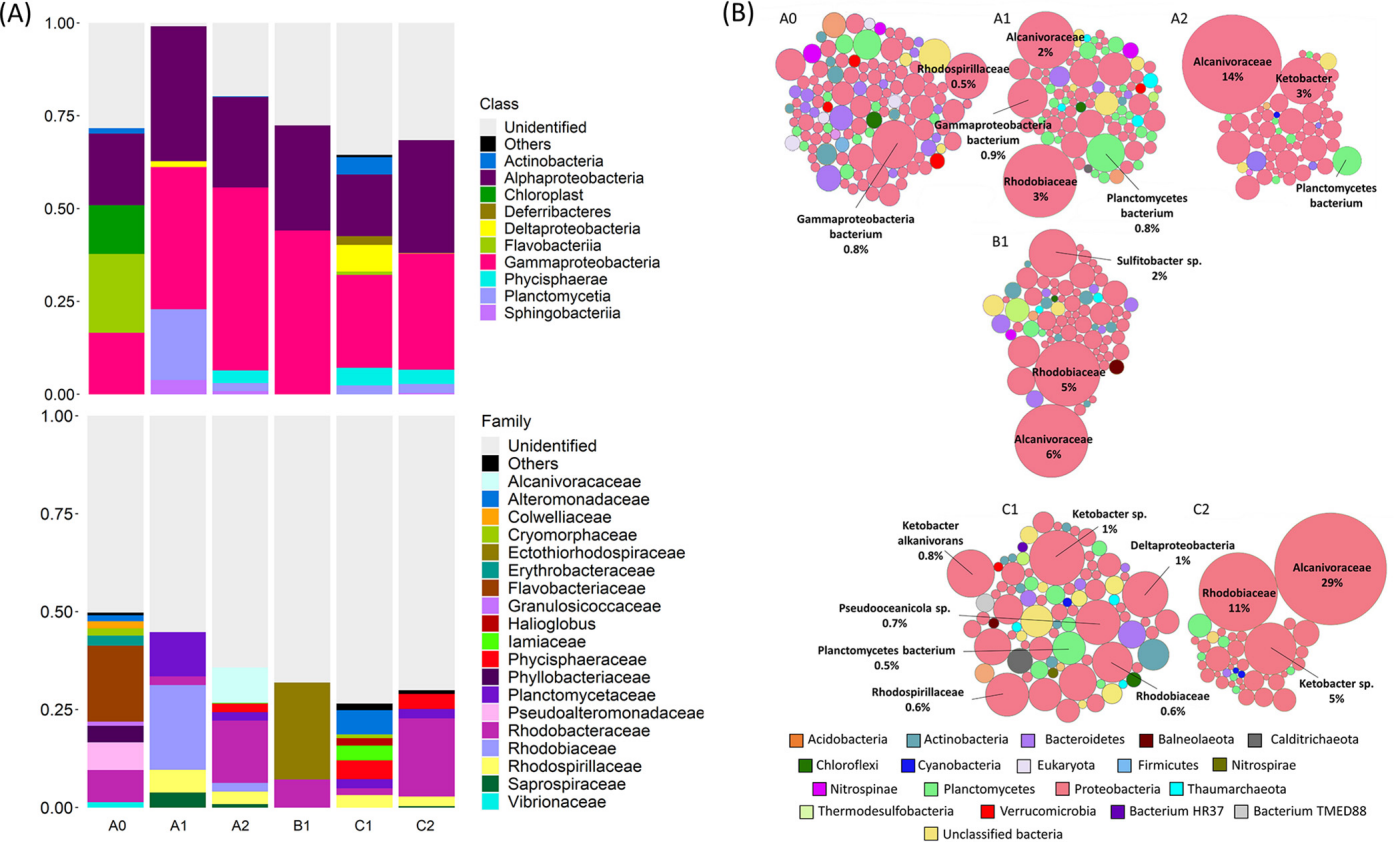
(A) Relative abundance of the abundant classified classes and families (>1% relative abundance) in the metagenomes obtained with matam. All classes and families with relative abundance below 1% were grouped in the category “Others.” (B) Bubble plot showing the relative taxonomic abundance of taxa that comprise at least 0.1% of classified reads in each sample. Bubble size indicates taxon abundance relative to the maximum abundance in the respective sample (largest bubble size per sample). Only the taxa with higher relative abundances are named. The numbers indicate the percentage of the total reads in each sample classified as the respective taxa.

Around 30%, 28%, 37%, 39%, 30%, and 59% of the reads of metagenomes A0, A1, A2, B1, C1, and C2, respectively, were taxonomically classified. Around 0.6% and 29% of the total number of reads in sample A0 were classified as eukaryotic and bacterial, respectively (Fig. 2). The number of reads classified as eukaryotic in all other samples was below 0.1%. *Alcanivoraceae*, *Ketobacter*, and *Rhodobiaceae* were the taxa with the highest relative abundance in samples after 2 years of incubations (Fig. 2). Analysis of the 16S rRNA gene assembled from the metagenomes revealed that after 2 years of incubation, communities A2 and C2 exhibited similar taxonomic compositions at the family level (Fig. 2), with *Phycisphaeraceae*, *Planctomycetaceae*, *Rhodobacteraceae*, *Rhodospirillaceae*, and *Saprospiraceae* representing similar relative abundances and constituting a major fraction of the classified families (Fig. 2). The *Rhodobacteraceae* family was the most abundant of all the identified families in communities A2 and C2, with relative abundances of 16% and 20%, respectively. *Rhodobacteraceae* were also abundant in community B1, with a relative abundance of 7%. It is nevertheless important to note that 55%, 64%, 68%, 73%, and 70% of the families classified with matam from communities A1, A2, B1, C1, and C2, respectively, remained unclassified.

**FIG 3:**
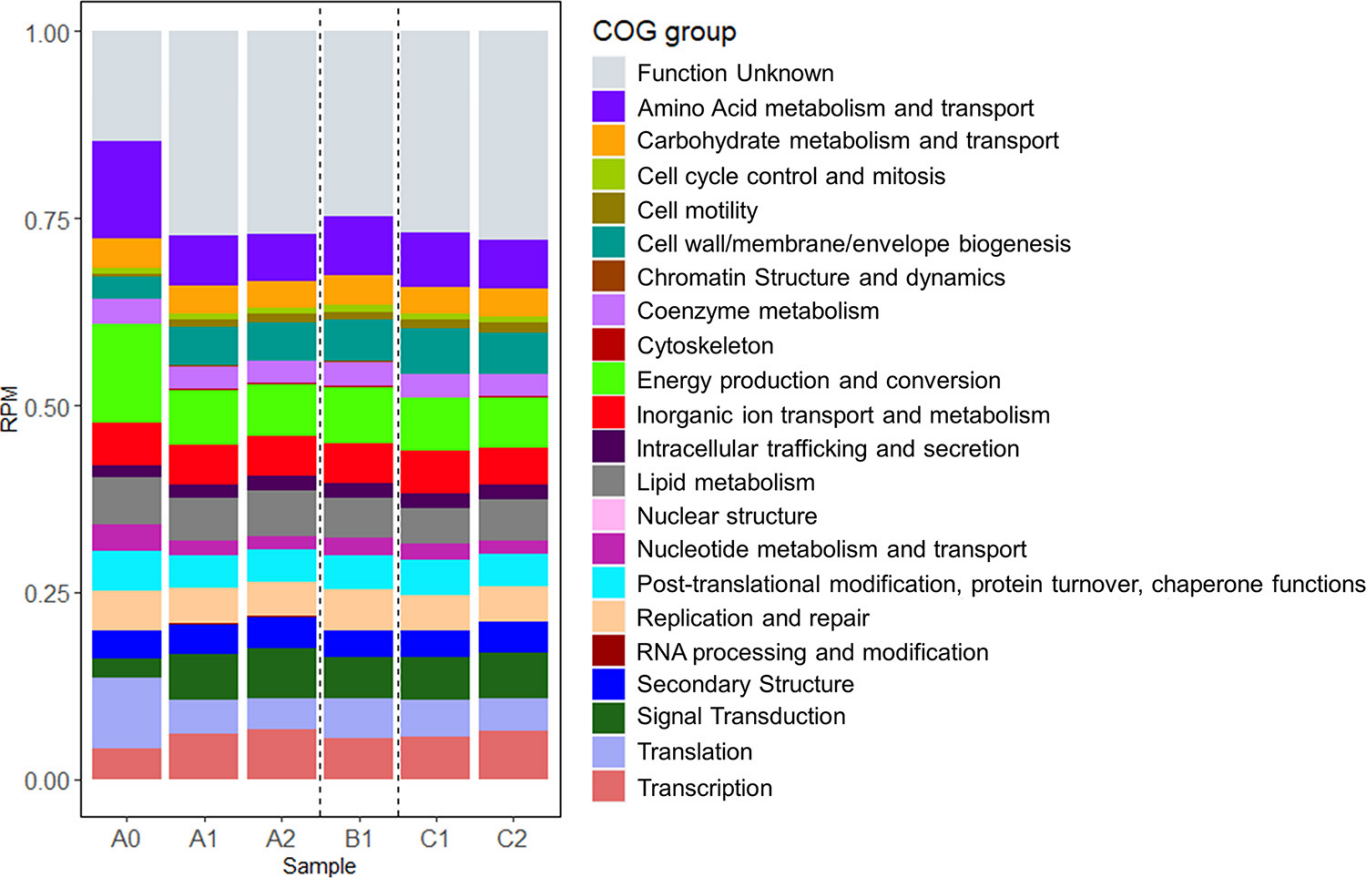
Relative abundance of the Clusters of Orthologous Groups (COG) functional groups in each sample, calculated from RPKM values.

Around 87%, 75%, 75%, 77%, 75%, and 74% of the predicted genes from samples A0, A1, A2, B1, C1, and C2, respectively, were assigned to a cluster of orthologous groups (COG) (Fig. 3). The main identifiable differences between the COG functional profiles of the different samples is the decrease in the relative abundance of genes assigned to amino acid metabolism and transport and to energy production and conversion in samples A0 to A1, accompanied by an increase of the relative abundance of genes assigned to signal transduction and transcription in the same sample. Samples A1, A2, B1, C1, and C2 exhibited relatively similar COG profiles.

### Phylogenetic classification of MAGs.

**FIG 4:**
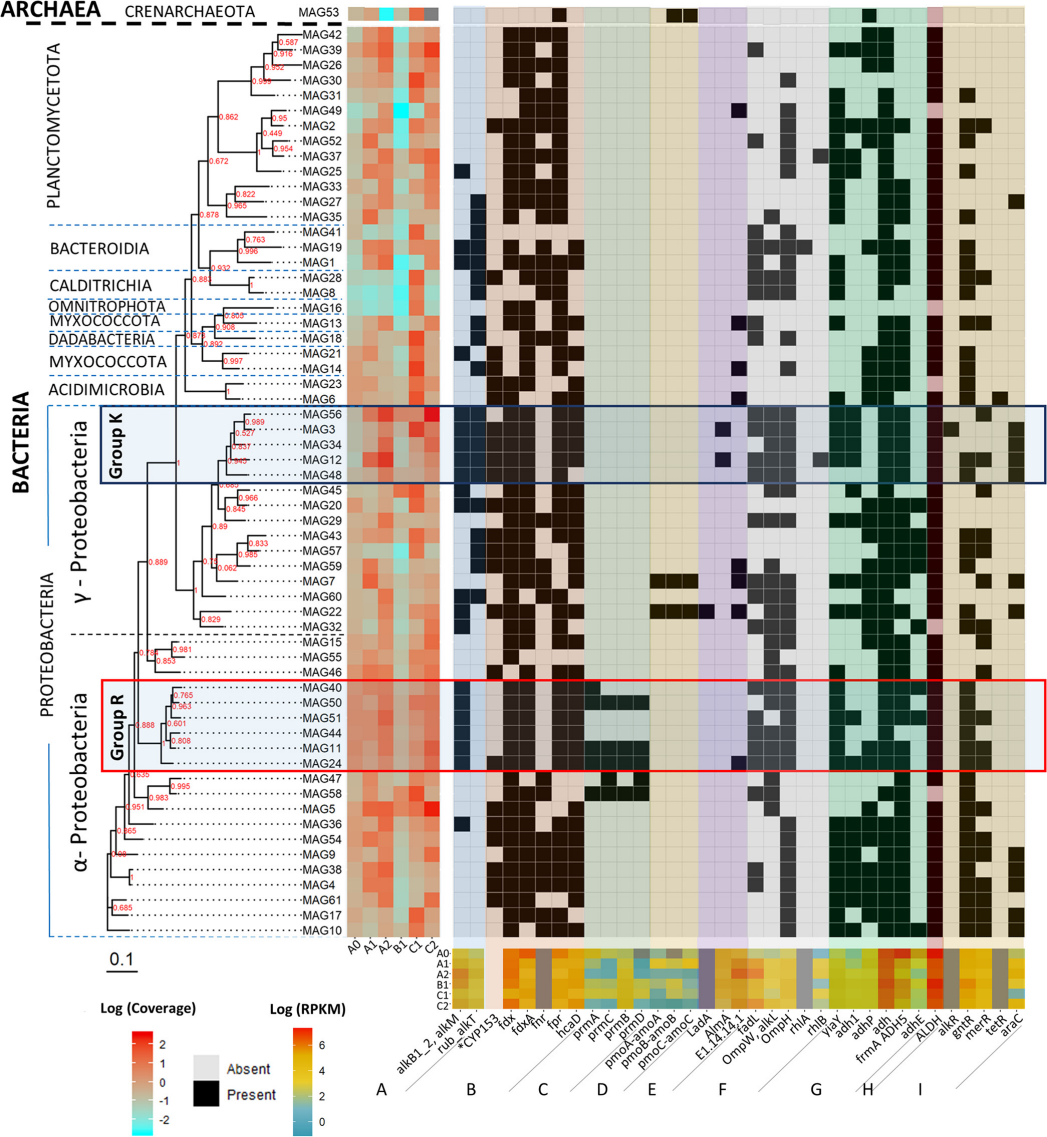
Phylogenetic tree of the identified MAGs, their coverage in the six metagenomes, and the presence or absence of selected alkane and/or fatty acid degradation related genes (Text S1) in each of the MAGs and their RPKM values in the different samples. The tree was built with Fasttree using the multiple-sequence alignment obtained with GTDB-tk. Bootstrap values of each tree branch are given in red. The different gene groups refer to genes related to the alkane 1-monooxygenase-encoding gene *alkB* (A), genes related to the cytochrome P450 alkane hydroxylase CYP153 (B), propane oxygenase-encoding genes (C), methane monooxygenase-encoding genes (D), genes encoding long-chain alkane-related monooxygenases and fatty acid oxygenases (E), transporter and biosurfactant synthesis genes (F), alcohol dehydrogenase genes (G), aldehyde dehydrogenase genes (H), and genes encoding proteins thought to be involved in transcription regulation of alkane degradation (I). Group K is a taxonomic clade, classified as *Pseudomonadales*, that includes the MAGs that had a high number of the selected genes and were closely related to *Ketobacter* and *Alcanivorax*. Group R includes all MAGs classified as *Rhodobacteraceae*, which all have the *alkB* gene, encoding the alkane monooxygenase most typically associated with alkane degradation. KEGG annotations were used to determine RPKM values of genes in different samples. *, the CYP153 gene does not have an assigned KO identifier; its presence/absence in MAGs was identified using HMMs.

From the 733,317 coassembled contigs, a total of 61 metagenome-assembled genomes (MAGs) with >50% completeness and <10% contamination were recovered (Table S1). The proportion of base pairs from the total reads of each sample assigned to the identified MAGs varied greatly between samples, with 3.3%, 58.9%, 78.5%, 24.6%, 73.2%, and 81.9% of the total base pairs of the metagenomes from communities A0, A1, A2, B1, C1, and C2, respectively, assigned to the base pairs of all 61 MAGs. Sixty of the MAGs were classified as bacterial and one as archaeal (MAG 53) (Fig. 4). The archaeal bin was classified as *Crenarchaeota* and was most abundant in community C1, but it was not found in C2 and was also present in very low relative abundances in communities B1 and A2 (Fig. 4 and Table S1).

10.1128/msystems.01415-21.8 TABLE S1 Information on obtained MAGs, their taxonomic classification, and coverage in each sample. Download Table S1, XLSX file, 0.02 MB. Copyright © 2022 Pinto et al. 2022 Pinto et al. https://creativecommons.org/licenses/by/4.0/ This content is distributed under the terms of the Creative Commons Attribution 4.0 International license.

10.1128/msystems.01415-21.1 TEXT S1 Information on selected genes encoding enzymes previously suggested to be involved in alkane and fatty acid biodegradation and list of bacterial strains associated with hydrocarbon and plastic degradation. The bacterial strains were selected if they followed at least one of the following criteria: they can survive with plastic or hydrocarbons as the sole carbon source, they are known to degrade alkanes and/or hydrocarbons, and they have been found associated with specific plastic types. Download Text S1, PDF file, 0.2 MB. Copyright © 2022 Pinto et al. 2022 Pinto et al. https://creativecommons.org/licenses/by/4.0/ This content is distributed under the terms of the Creative Commons Attribution 4.0 International license.

**FIG 5:**
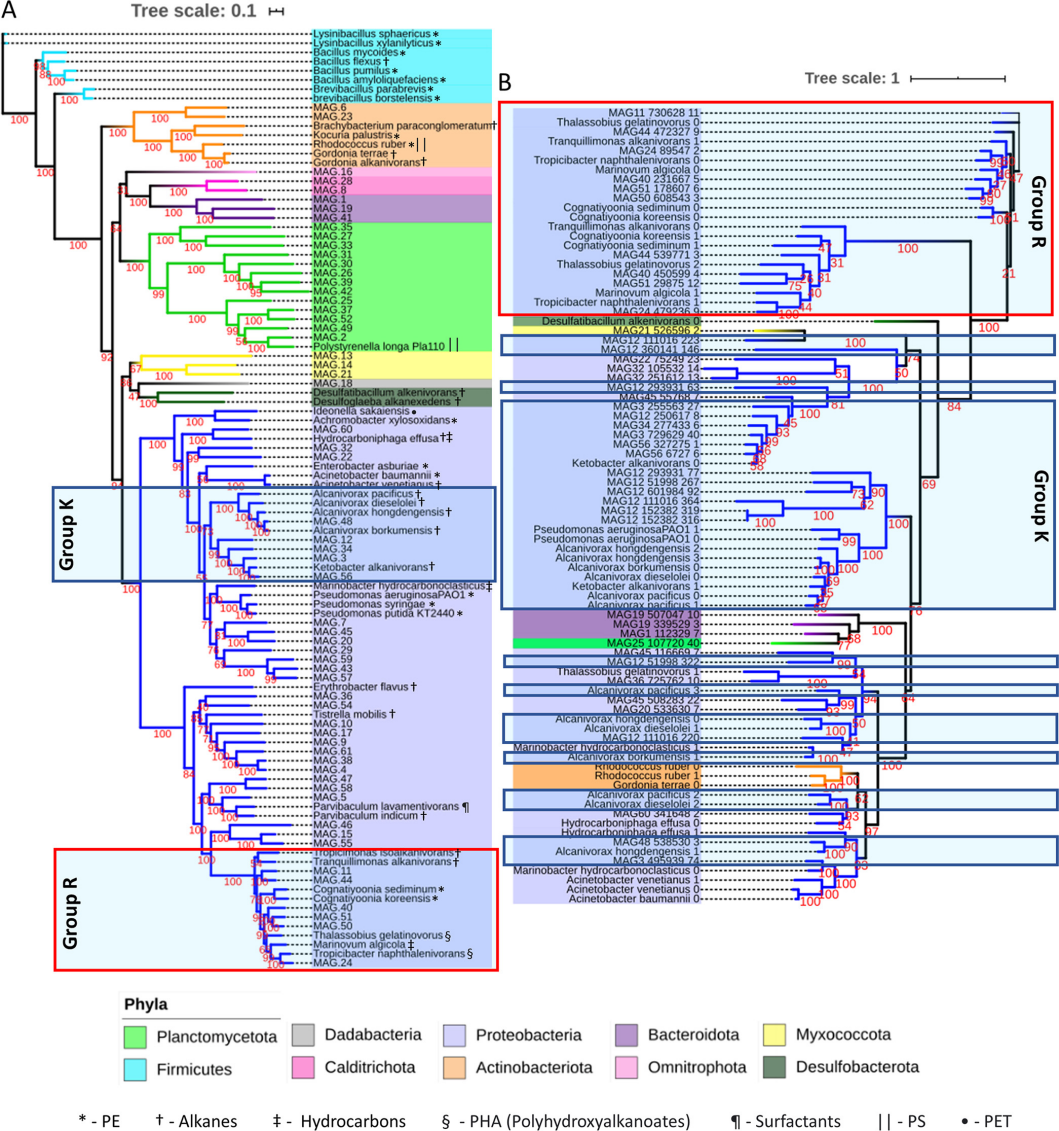
Phylogenetic placement of the MAGs and selected genomes (Text S1) (A) and putative *alkB* sequences identified in MAGs and genomes (B). Bootstrap values are indicated in red. Group K (blue boxes) and Group R (red boxes) refer to the same groups as described in the legend for Fig. 2. The symbols in the tree in panel A represent the type of substrate the corresponding strain was associated with. For more detailed information, see Text S1.

MAGs 5 and 56, belonging to the family *Parvibaculaceae* and genus *Ketobacter*, respectively, increased in coverage in both communities A and C from the first to the second year and were among the MAGs with the highest coverage in the communities A2, B1, and C2 (Fig. 4 and Table S1). We compared the obtained MAGs with the genomes of potential hydrocarbon, alkane, or plastic degraders (Text S1) retrieved from the NCBI (Fig. 5A). MAGs 5 and 56 clustered together with alkane-degrading species of the genus *Parvibaculum* and the alkane degrader Ketobacter alkanivorans, respectively (Fig. 5A). MAG 56 was grouped in a larger cluster (group K in Fig. 4 and 5) composed of MAGs classified as *Alcanivoraceae* and *Ketobacteraceae* and the genomes of known *Alcanivorax* species and *Ketobacter alkanivorans* (Fig. 5). Generally, MAGs from group K increased over time (Fig. 4). Other MAGs also increased in coverage over time in communities A and C (MAGs 1, 13, 15, 26, 27, 37, 39, 42, 44, 49, 51, 60, and 61) (Fig. 4). However, a differential abundance analysis determined that only MAGs 9, 11, 15, 24, 29, 32, 34, 56, 60, and 61 had genes that were significantly enriched after 2 years of incubation in comparison to the 1-year incubations in communities A and C (Table S2).

10.1128/msystems.01415-21.9 TABLE S2 Result of DESeq2 analysis, presenting genes that were significantly enriched after 2 years of incubation in comparison to the 1-year incubations in communities A and C, and their respective MAGs (sheet 1). RPKM values are shown for all genes that increased in RPKM over time (sheet 2), and for enriched genes encoding oxidoreductases (sheet 3), in both A and C samples. The genes are ordered by their mean RPKM values, from most abundant on top to least abundant. Download Table S2, XLSX file, 0.2 MB. Copyright © 2022 Pinto et al. 2022 Pinto et al. https://creativecommons.org/licenses/by/4.0/ This content is distributed under the terms of the Creative Commons Attribution 4.0 International license.

Most MAGs exhibited a very low coverage in community B1, with the exception of some classified as *Proteobacteria*, which is also supported by the 16S rRNA gene analysis (Fig. 2). MAGs 5, 58, 45, and 56 were the only ones with coverage values higher than 1 in community B1, with values of 3.93, 2.95, 12.57, and 5.11, respectively.

### Functional gene dominance in the metagenome over time.

A total of 460 genes were significantly enriched (Table S2) after 2 years of incubation in comparison to the 1-year incubations in communities A and C as determined by a differential abundance analysis. Of these genes, 10, 108, 4, 17, 10, 2, 204, 1, 21, and 2 were, respectively, assigned to MAGs 9, 11, 15, 24, 29, 32, 34, 56, 60, and 61. The remaining 81 genes were not assigned to any MAG.

A DESeq2 analysis of all genes when grouped in KEGG orthologs (KO numbers) showed a significant differential abundance (*P* value < 0.05) of certain selected genes (Text S1), namely, genes *araC* and *pmoA-amoA*, encoding the AraC family transcriptional regulator and subunit A of the methane/ammonia monooxygenase, respectively, and genes encoding a propane 2-monooxygenase (*prmA*, *prmC*, and *prmD*) between 1 year of incubation and 2 years of incubation in communities A and C. These genes all decreased in relative abundance over time in both communities.

To determine which metabolic pathways increased in the incubations over time, we compared the predicted genes with RPKM (reads per kilobase million) values that increased from the original community to the community after 1 year of incubation in the case of community A and from 1 to 2 years of incubation in both communities A and C. Predicted genes annotated as glutathione *S*-transferase (GST) homologues were overall the most abundant genes increasing in RPKM over time, even though they were already present in fairly high relative abundance in the original sample A0 (Table S2). GST homologue genes are involved in polycyclic aromatic hydrocarbon (PAH) degradation in prokaryotes, as well as in the biodegradation of other xenobiotics (34). Other genes that increased in RPKM values over time in the LDPE treatments, like *atzF* and *mlhB*, are also involved in xenobiotic degradation.

A large part of the oxidoreductase-encoding genes increasing in RPKM in communities A2 and C2 were annotated as genes related to lipid metabolism, especially fatty acid and steroid metabolism (Table S2). Furthermore, all the identified complete KEGG pathway modules with increasing RPKM values were related to lipid metabolism, particularly fatty acid biosynthesis, fatty acid beta-oxidation with the synthesis of acyl-CoA, and acylglycerol and sphingosine degradation. The CYP51 gene (annotated by KEGG) was the gene of the oxidoreductase category that increased most pronouncedly in RPKM in communities A2 and C2 (Fig. S2). It encodes sterol 14 alpha-demethylase, a cytochrome P450 enzyme which plays an important role in sterol synthesis and also in fatty acid metabolism (35). Another gene, cypD_E, also significantly increased in RPKM over time, especially in community C2 (Fig. S2). cypD_E encodes cytochrome P450/NADPH-cytochrome P450 reductase, linked to fatty acid oxidation. It catalyzes the hydroxylation of a range of long-chain fatty acids and oxidizes NADPH by electron transfer to the heme iron of the cytochrome P450 N-terminal domain (36).

10.1128/msystems.01415-21.4 FIG S2 Plot showing the RPKM values (in log) of genes encoding KEGG-annotated oxidoreductases and in all six metagenomes. Download FIG S2, TIF file, 0.8 MB. Copyright © 2022 Pinto et al. 2022 Pinto et al. https://creativecommons.org/licenses/by/4.0/ This content is distributed under the terms of the Creative Commons Attribution 4.0 International license.

Two predicted genes, annotated as monooxygenases involved in fatty acid degradation, also increased in RPKM over time in communities A2 and C2. One of the predicted genes was classified as encoding a cytochrome P450/NADPH-cytochrome P450 reductase monooxygenase, and the other gene was annotated as gene alkB1_2, encoding an alkane 1-monooxygenase involved in alkane degradation (Fig. 1 and Fig. S2). Alkane 1-monooxygenases catalyze the hydroxylation of *n*-alkanes and fatty acids in the presence of NADH-rubredoxin reductase and rubredoxin. A gene annotated as *rub*, encoding the rubredoxin-NAD(+) reductase, was also among the genes that increased in relative abundance over time (Fig. 4 and Table S3). The rubredoxin-NAD(+) reductase transfers electrons from NADH to rubredoxin reductase and further through rubredoxin to alkane 1-monooxygenase.

10.1128/msystems.01415-21.10 TABLE S3 Tukey test results comparing the cell abundance (cells cm^−1^) between different pairs of samples. Significant differences (*) were determined at a *P* value of <0.05 (sheet 1). Tukey test results comparing the total oxygen consumption (μmol O_2_ cm^−2^) and the oxygen consumption rates (μmol O_2_ h^−1^ cell^−1^) between different pairs of samples are shown. Significant differences (*) were determined at a *P* value of <0.05 (sheet 2). Download Table S3, XLSX file, 0.01 MB. Copyright © 2022 Pinto et al. 2022 Pinto et al. https://creativecommons.org/licenses/by/4.0/ This content is distributed under the terms of the Creative Commons Attribution 4.0 International license.

There were several other genes increasing over time encoding enzymes related to fatty acid metabolism. Some of these genes, i.e., genes encoding lipid and fatty acid transporters (genes *lip*, *fadL*, and *ompW*) and alcohol (genes *yiaY* and *adh1*) and aldehyde (gene ALDH7A1) dehydrogenases (Table S2) (30) are the key genes in the alkane degradation pathway.

### Putative hydrocarbon degradation genes in MAGs.

Overall, most MAGs that increased in coverage over time either showed the potential to degrade alkanes or had genes encoding alkane and hydrocarbon transporters. MAGs 3, 12, and 22, classified as *Gammaproteobacteria*, had at least one medium-length alkane oxidation-related gene and either the AlmA or the LadA gene, encoding enzymes potentially degrading both long- and medium-chain alkanes (Fig. 4). MAG 22 also had all three genes encoding a methane/ammonia monooxygenase, suggesting that this organism is also capable of degrading short-chain alkanes. Furthermore, these MAGs also had at least one gene encoding a surfactant and alkane membrane transporters. Group K MAGs, classified as *Pseudomonadales* (shown in Fig. 4), all had genes encoding aldehyde and alcohol dehydrogenases, at least two alkane-related transporters, and proteins associated with surfactant production. They also harbored the alkB1_2 and *alkT* genes, encoding alkane monooxygenase and rubredoxin-NAD^+^ reductase, respectively, which together form a complex capable of oxidizing medium-chain alkanes (Fig. 4). Group K MAGs also harbored genes encoding a cytochrome P450 alkane monooxygenase (CYP153), the associated transcription regulator gene *araC*, and the genes encoding a ferredoxin and ferredoxin reductase, which are required for the transfer of electrons from NAD(P)H to the cytochrome (37).

MAG 5, classified as belonging to the *Parvibaculaceae* family, which was among the most abundant MAGs in community C2, harbored a gene encoding a protein associated with surfactant production and alkane transport into the cell (OmpW [AlkL]). MAGs 24, 11, 44, 51, 50, and 40, which together formed the *Rhodobacteraceae* clade (group R in Fig. 4), all had genes annotated as alkB1_2, *hcaD*, and the surfactant producing membrane protein-encoding gene *ompH*. No rubredoxin reductase-encoding gene was annotated in the group R MAGs. However, they all had genes encoding ferredoxin and ferredoxin reductase. It has been suggested that ferredoxin and ferredoxin reductase might replace rubredoxin and rubredoxin reductase, usually associated with *alkB* (38). MAG 58, classified as belonging to the *Methyloligellaceae* family, together with three of the group R MAGs also harbored the four genes encoding the different subunits (Fig. 4, *prmABCD*) of an oxidoreductase that oxidizes propane (propane 2-monooxygenase), a three-carbon alkane that is a gas at standard temperature and pressure.

Furthermore, group R MAGs, except MAG 51, classified as a *Pseudooceanicola* sp., harbored the gene *ompW* (*alkL*) encoding an outer membrane protein, involved in 1-alkane transport into the cell and surfactant production. MAG 24, classified as a *Sagittula* sp., also had a gene encoding a cytochrome P450/NADPH-cytochrome P450 reductase monooxygenase. With the exception of MAG 50, all group R MAGs harbored the gene *fadL*. Almost all MAGs harbored at least one aldehyde dehydrogenase- and one transcriptional regulator-encoding gene, both involved in several metabolic processes within the cell.

### Distribution of the *alkB* and CYP153 genes in the MAGs.

Hidden Markov models (HMMs) were used to identify putative *alkB* and CYP153 genes in the MAGs and the genomes of potential hydrocarbon and plastic degraders (Text S1). Totals of 21 and 23 MAGs had at least one copy of genes classified as *alkB* and CYP153, respectively (Fig. 4). *alkB* was found in 1 *Myxococcota* and 1 *Planctomycetota* MAG, in 2 *Bacteroidota* MAGs, and in 17 *Proteobacteria* MAGs. Multiple copies of the *alkB* gene were found in some of the MAGs, especially in MAGs from group K. The highest number of *alkB* genes in one MAG was 12, found in MAG 12 (Fig. 5B). *alkB* genes belonging to group R MAGs clustered together in one single group. The *alkB* genes from group R MAGs clustered with the *alkB* genes from known marine *Rhodobacteraceae* alkane degraders (Fig. 5B). In contrast, *alkB* genes from group K clustered together in several groups, with some of them grouping with sequences from other *Proteobacteria* MAGs (Fig. 5B).

CYP153 was detected in 1 MAG of *Calditrichota* and of *Planctomycetota*, in 2 MAGS of *Actinobacteriota* and of *Myxococcota*, and in 17 MAGs classified as *Proteobacteria*. There were several clusters composed only of *Proteobacteria* CYP153; however, there were also CYP153 genes from MAGs of different phyla clustering together (Fig. S3). There were several MAGs with more than one copy of a gene classified as CYP153 (Fig. S3).

10.1128/msystems.01415-21.5 FIG S3 Phylogenetic placement of the MAGs and selected genomes (A) and putative CYP153 sequences identified in MAGs and genomes (B). Download FIG S3, TIF file, 1.6 MB. Copyright © 2022 Pinto et al. 2022 Pinto et al. https://creativecommons.org/licenses/by/4.0/ This content is distributed under the terms of the Creative Commons Attribution 4.0 International license.

### Cell abundance and respiration measurements.

**FIG 6:**
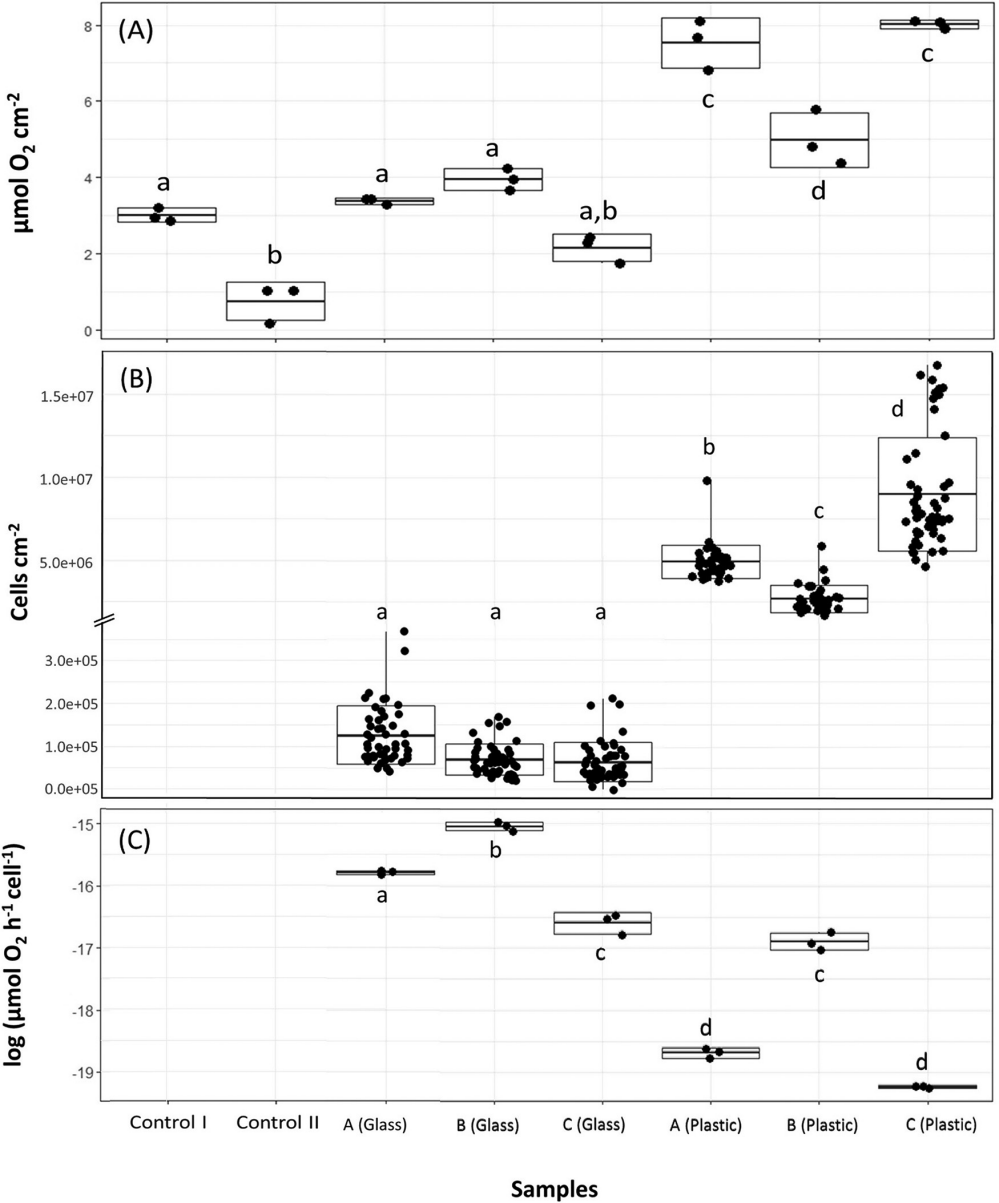
Oxygen consumption rates of microbes in the plastic and glass treatments. The boxplots represent the means ± SD. (A) Total oxygen consumption in each sample in micromoles per square centimeter of plastic or glass. Each black dot represents one replicate. (B) Number of cells per square centimeter of plastic or glass. In this plot, the boxplot lines represent the maximum number of cells cm^−2^ counted in a photo of that sample. Each black dot corresponds to the number of cells per square centimeter of one photo. The controls were excluded from this and the next plot because no cells in those samples were detected. (C) Oxygen consumption. Samples with different letters on each plot were significantly different from each other (*P* value < 0.05; Tukey test results in Table S3).

To determine respiration rates of the biofilms on plastic in comparison to glass, oxygen consumption measurements were performed after the surfaces were incubated for 3 months with communities A2, B2, and C2 (Fig. 1). Just before starting the respiration measurements, the abundances of all three microbial communities were significantly higher in the incubations with plastics than with glass (*F* = 955.18 and *P* < 0.001) (Fig. 6B and Fig. S4). The cell abundances of the three communities growing on LDPE also differed between each other (*F* = 96.41 and *P* < 0.001) (Table S3), with community C exhibiting a significantly higher number of cells than communities A and B (Fig. 6). No cells were found on the surface of both LDPE controls (control I and control II).

10.1128/msystems.01415-21.6 FIG S4 Fluorescence microscope photos of DAPI-stained cells on plastic and glass. Download FIG S4, TIF file, 1.6 MB. Copyright © 2022 Pinto et al. 2022 Pinto et al. https://creativecommons.org/licenses/by/4.0/ This content is distributed under the terms of the Creative Commons Attribution 4.0 International license.

Oxygen consumption rates per square centimeter of surface area were significantly higher in the microbial communities associated with LDPE than on glass (*F* = 48.84 and *P* < 0.001) and in the controls (Fig. 6A and Table S3). However, cell-specific oxygen consumption rates were higher in microbial communities in the glass than in the plastic treatment (Fig. 6C). Incubations with community B2 showed the least difference in bulk oxygen consumption rates between the glass and plastic treatments. Additionally, from all the treatments with LDPE, community B2 exhibited the highest cell-specific oxygen consumption rate. Communities A2 and C2 showed similar cell-specific oxygen consumption rates (Fig. 6C) (*P* = 0.99). However, when incubated with glass, community A2 showed a higher oxygen consumption rate than community C2 (Fig. 6C) (*P* < 0.001).

### Alteration of plastic surfaces determined by ATR-FTIR, SEM, and weight.

**FIG 7:**
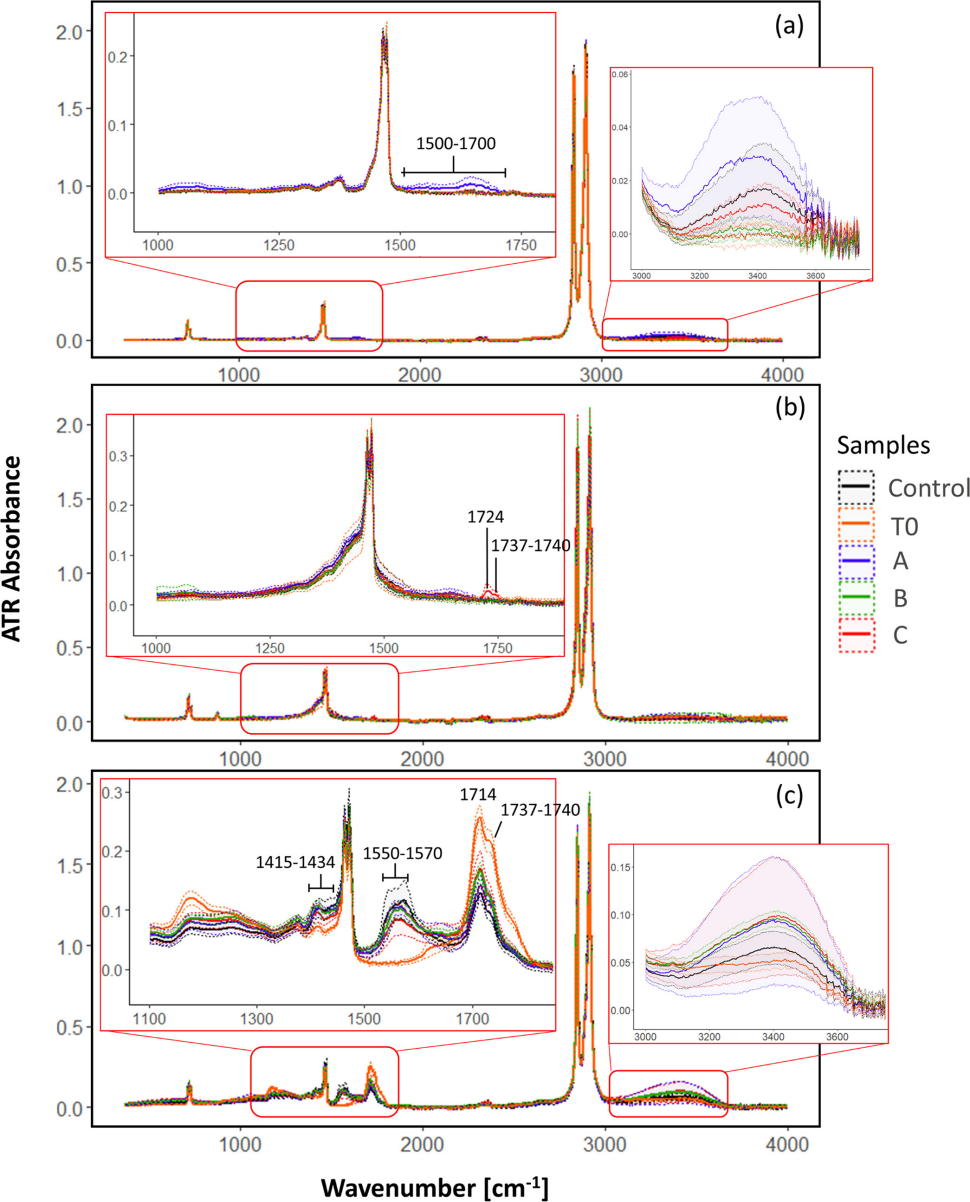
ATR-FTIR spectra of nonweathered LDPE sheets (a), packaging PE sheets (b), and UV-weathered LDPE sheets (c) prior to the incubation and after 6 months of incubation with the three microbial communities. The lines correspond to the mean values of the replicates, and dashed lines of the same color represent the corresponding standard deviation.

The ATR-FTIR spectra of the untreated LDPE sheets, packaging LDPE, and UV-treated LDPE sheets (prior to microbial incubations) before and after 6 months of exposure to microbial communities growing previously on PE as the sole carbon and energy source for 2 years are depicted in Fig. 7. All plastics shared the typical LDPE bands for CH_3_ and –CH_2_– groups at 2,916, 2,849, 1,469, 1,375, and 718 cm^−1^.

**FIG 8:**
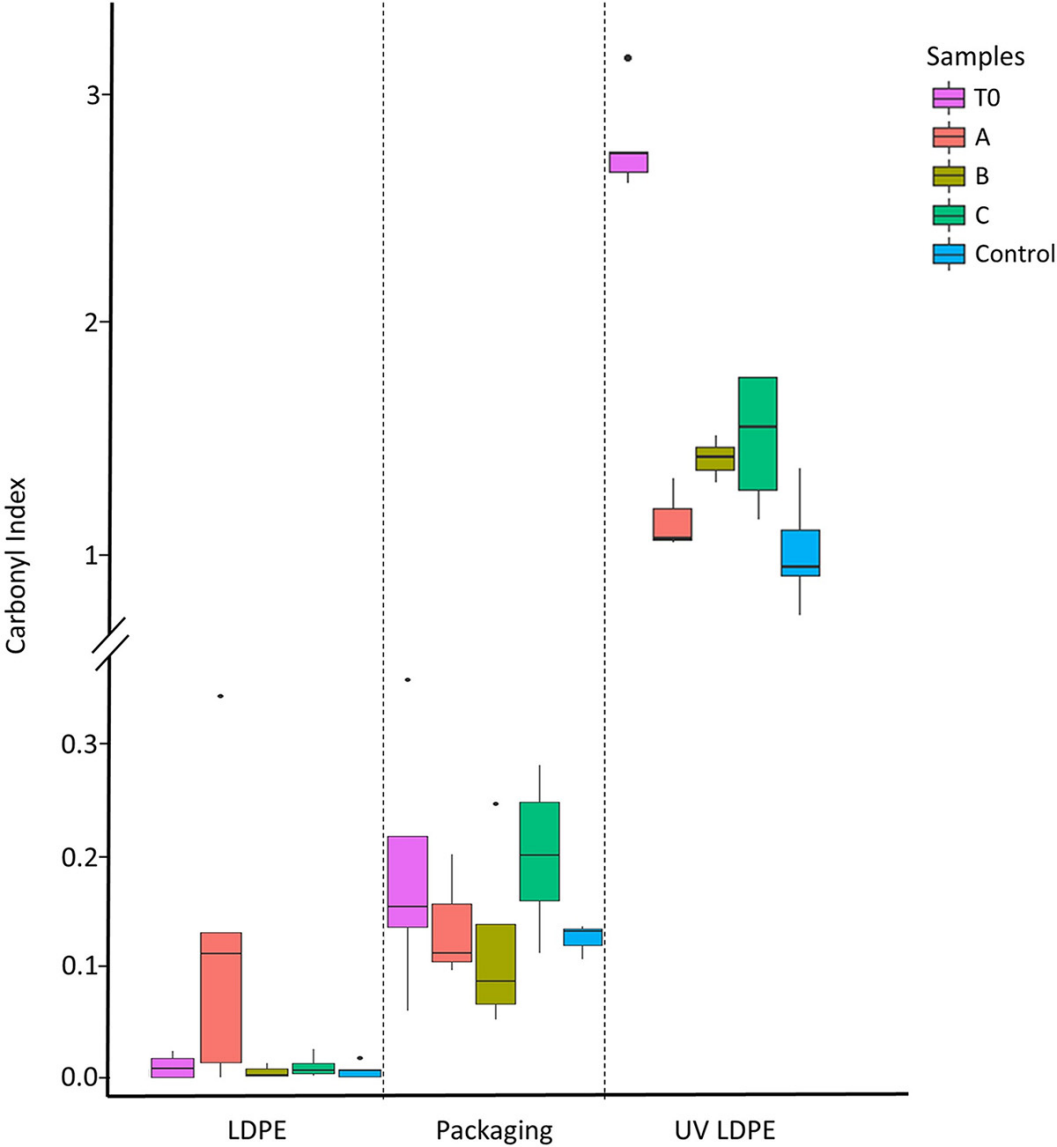
Carbonyl indices of the different sample types, incubated with different communities. The boxplots represent the median, the first and third quartiles, and the 95% confidence interval of the median. The dots represent outliers.

In nonweathered LDPE plastics incubated with community A, a band spreading from 1,500 to 1,700 cm^−1^ appeared, indicating the presence of carbonyl (>C = O) and vinyl (–CH=CH–) groups (Fig. 7). Also, a small peak at 3,400 cm^−1^ was detected, which might indicate the appearance of hydroxyl groups. Bands at around 1,640 and 3,400 cm^−1^, however, might also indicate the presence of water. The spectra from packaging PE pieces incubated with community C revealed peaks at 1,724 and at 1,737 to 1,740 cm^−1^, corresponding to the appearance of carbonyl groups, carboxylic groups, and aldehydes, respectively. At the beginning of the incubations, UV-weathered plastics exhibited an additional band at 1,714 cm^−1^, corresponding to the appearance of carbonyl groups resulting from photooxidation of polyethylene. After 6 months of incubation, peaks indicating carboxylates appeared at 1,550 to 1,570 cm^−1^ and 1,415 to 1,434 cm^−1^ in all incubations. The peak at 1,714 cm^−1^ had higher intensity in UV-weathered plastics incubated with microbes than in the control samples without microbes, but it was of lower intensity than prior to the incubation experiment. A small peak at about 3,400 cm^−1^ also appeared, especially in the plastics incubated with bacteria. There were also signs of oxidation in the control treatment without microbes of the UV-weathered LDPE. This degradation might be the result of the presence of inorganic ions working as catalysts of oxidation of the carbonyl compounds to carboxylic compounds (39). To evaluate differences in oxidation states of the different plastics, the carbonyl index (CI) for each sample was calculated (Fig. 8). UV-treated LDPE presented significantly higher CI values (*P* < 0.05) (Table 1) when incubated with community C than the control samples. Interestingly, T0 UV-treated LDPE samples had considerably higher CI values than the control (Table 1) and similar pieces incubated with the microbial communities. This could be due to a reaction of the highly oxidized UV-treated LDPE with the artificial seawater, which might also be related to the appearance of the bands at 1,550 to 1,570 cm^−1^ in the non-T0 samples.

**TABLE 1:** ANOVA results comparing CIs from the different plastics incubated under different treatments^*a*^

Samples	LDPE UV				Packaging				LDPE			
	Diff	Lwr	Upr	*P*_adj_	Diff	Lwr	Upr	*P*_adj_	Diff	Lwr	Upr	*P*_adj_
B-A	2.7E−01	−2.6E−01	7.9E−01	5.6E−01	−1.8E−02	−2.0E−01	1.7E−01	1.0E+00	−1.1E−01	−2.7E−01	4.0E−02	2.1E−01
C-A	3.5E−01	−1.2E−01	8.2E−01	2.0E−01	6.4E−02	−1.1E−01	2.4E−01	7.8E−01	−1.1E−01	−2.5E−01	3.3E−02	1.8E−01
Control-A	−1.4E−01	−5.7E−01	3.0E−01	8.7E−01	−1.2E−02	−2.1E−01	1.9E−01	1.0E+00	−1.1E−01	−2.5E−01	2.1E−02	1.2E−01
T0-A	1.6E+00	1.2E+00	2.1E+00	0.0E+00*	4.8E−02	−1.3E−01	2.3E−01	9.2E−01	−1.1E−01	−2.5E−01	3.2E−02	1.7E−01
C-B	8.8E−02	−3.8E−01	5.6E−01	9.8E−01	8.2E−02	−7.5E−02	2.4E−01	5.1E−01	4.8E−03	−1.6E−01	1.7E−01	1.0E+00
Control-B	−4.0E−01	−8.4E−01	3.2E−02	7.7E−02	6.8E−03	−1.8E−01	1.9E−01	1.0E+00	1.2E−03	−1.5E−01	1.6E−01	1.0E+00
T0-B	1.4E+00	9.0E−01	1.8E+00	4.0E−07*	6.6E−02	−9.7E−02	2.3E−01	7.3E−01	4.6E−03	−1.6E−01	1.7E−01	1.0E+00
Control-C	−4.9E−01	−8.6E−01	−1.2E−01	5.7E−03*	−7.6E−02	−2.5E−01	9.7E−02	6.7E−01	−3.6E−03	−1.5E−01	1.4E−01	1.0E+00
T0-C	1.3E+00	8.7E−01	1.7E + 00	1.0E−07*	−1.6E−02	−1.6E−01	1.3E−01	1.0E + 00	−2.1E−04	−1.5E−01	1.5E−01	1.0E+00
T0-control	1.8E+00	1.4E+00	2.1E+00	0.0E+00*	5.9E−02	−1.2E−01	2.4E−01	8.4E−01	3.4E−03	−1.4E−01	1.5E−01	1.0E+00

a Diff, difference; Lwr, lower; Upr, upper; *P*_adj_, adjusted *P* value. *, significant differences (*P* value < 0.05) between the CI of the compared samples.

**FIG 9:**
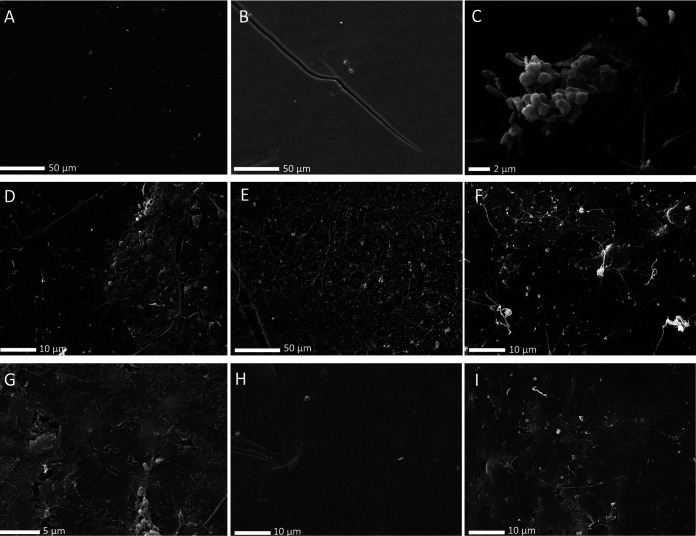
SEM images of samples collected after 3 months of incubation with the three different communities. (A) LDPE control sheet; (B) UV-weathered LDPE control; (C) LDPE sheet incubated with community C2; (D) LDPE sheet incubated with community A2; (E) UV-weathered LDPE incubated with community C2; (F) packaging PE incubated with community C2; (G) LDPE sheet incubated with C2, after biofilm removal; (H) UV-weathered LDPE sheet incubated with B1, after biofilm removal; (I) packaging PE incubated with C2 after biofilm removal.

After 3 months of incubation a visible biofilm developed on all PE samples, except in the controls (Fig. 9A to F). In some samples, after removal of the biofilm, there were some visible holes and apparent changes in the plastic surface, especially in the previously UV-weathered LDPE and packaging PE (Fig. 9G to I) when incubated with communities A and C.

**FIG 10:**
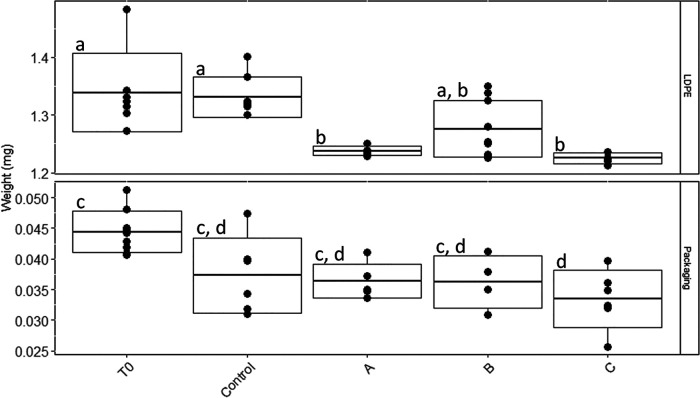
Weights of nonweathered LDPE and packaging PE pieces incubated with the three different communities A, B, and C for 8 months. The boxplot represents the mean ± SD, with the whiskers representing the minimum and maximum weight values. Each dot represents the weight of an individual plastic piece. Different letters within each panel refer to samples with statistically significant differences (*P* value < 0.05) (Table 2).

Significant weight losses were found between different treatments and/or time points in the packaging PE and nonweathered LDPE (*F* = 6.847 and *P* < 0.001 and *F* = 9.34 and *P* < 0.001, respectively). Nonweathered LDPE plastics significantly decreased in weight over a period of 8 months of incubation compared to the controls without bacteria (Fig. 10 and Table 2). The loss in weight over the 8 months amounted to 7.5%, 5%, and 8.5% in plastics incubated with communities A, B, and C, respectively. All PE packaging plastics, including the control samples, significantly decreased in weight after 8 months of incubation (Fig. 10).

**TABLE 2:** Tukey test results comparing the weights of the packaging PE and nonweathered LDPE incubated with the three different microbial communities^*a*^

Samples	Nonweathered LDPE				Packaging PE			
	Diff	Lwr	Upr	*P*_adj_	Diff	Lwr	Upr	*P*_adj_
Control-T0	−0.008	−0.073	0.057	0.997	−0.007	−0.014	0.000	0.035*
A-T0	−0.101	−0.175	−0.027	0.003*	−0.008	−0.015	−0.001	0.021*
B-T0	−0.063	−0.126	0.001	0.053	−0.008	−0.016	−0.001	0.032*
C-T0	−0.114	−0.184	−0.044	0.000*	−0.011	−0.018	−0.004	0.001*
A-control	−0.093	−0.165	−0.022	0.006*	−0.001	−0.009	0.007	0.996
B-control	−0.055	−0.116	0.006	0.094	−0.001	−0.009	0.007	0.995
C-control	−0.106	−0.174	−0.038	0.001*	−0.004	−0.011	0.004	0.556
B-A	0.038	−0.032	0.108	0.520	0.000	−0.009	0.008	1.000
C-A	−0.012	−0.089	0.064	0.989	−0.003	−0.011	0.005	0.804
C-B	−0.051	−0.117	0.016	0.201	−0.003	−0.011	0.005	0.862

a Significant differences (*) were determined at a *P* value of <0.05.

## DISCUSSION

Our results suggest that LDPE-associated compounds can serve as a carbon and energy source for at least a part of the plastic biofilm community. We have also identified several organisms, genes, and associated pathways related to alkane and fatty acid metabolism that might be involved in the utilization of LDPE and its associated compounds as a carbon source.

Even though the three communities were derived from three different plastics and even a different season in the case of community C, there were several similarities in taxonomy and functional diversity between communities A and C after 2 years of incubation. The original community A0 was dominated by families typically found in plastic biofilms, such as *Flavobacteriaceae*, *Pseudoalteromonaceae*, *Alteromonadaceae*, and *Rhodobacteraceae* (Fig. 2) (40). Over time, the families *Flavobacteriaceae*, *Pseudoalteromonaceae*, and *Alteromonadaceae* decreased in relative abundance, while the family *Rhodobacteraceae* dominated the classified families in communities A2 and C2 (Fig. 2).

The two MAGs with the highest coverage in the A2 and C2 communities, classified as *Parvibaculaceae* and *Ketobacter* (MAGs 5 and 56, respectively), are closely related to species known to be involved in alkane degradation (Fig. 5A). *Ketobacter alkanivorans* is a recently characterized marine *n*-alkane degrading bacterium and, until now, the only classified species of the *Ketobacter* genus (41). Even though in the recently proposed rank-normalized Genome Taxonomy Database (GTDB) taxonomy (42) *Ketobacter* belongs to the *Ketobacteraceae* family, it was previously grouped together with the genus *Alcanivorax* in the *Alcanivoraceae* family. *Alcanivorax* and closely related bacteria have been shown to thrive after oil spills (43) and dominate petroleum-contaminated waters when adequate amounts of nitrogen and phosphorus are available (44). This is mainly attributed to their high ability to degrade alkanes. In our incubations, *Ketobacteraceae* and *Alcanivorax* were among the MAGs with the highest increase in relative abundance over time, and they have the genetic potential for alkane degradation. While the role of alkane degradation in LDPE degradation is unclear, our results further suggest that if the LPDE polymer can in fact be degraded, alkane-related metabolism might be involved.

The family *Parvibaculaceae* (previously *Rhodobiaceae*), to which MAG 5 belongs, comprises species from the genus *Parvibaculum* known for their ability to degrade polycyclic aromatic hydrocarbons and laundry detergents (45–47). Additionally, some of the other relatively abundant MAGs in communities A and C after 1 and 2 years of incubation, like the ones classified as *Acidimicrobia* (previously *Actinobacteria*) and *Myxococcota* (previously order *Myxococcales*), have also been found in hydrocarbon-polluted areas and associated with alkane degradation (48). These MAGs also harbored essential genes encoding enzymes for alkane degradation. The fact that several of the most abundant MAGs in communities A2, B1, and C2 were identified as close relatives of hydrocarbon degraders and harbored genes encoding all essential enzymes for alkane degradation is additional evidence that hydrocarbon-degrading organisms were positively selected in our incubations. This supports the hypothesis that alkane degradation pathways might be used in the degradation of LDPE-associated compounds. While *Actinobacteriota* and several alkane-degrading *Proteobacteria*, like those belonging to the *Alcanivorax* genus or the *Rhodobacteraceae* family, have already been identified and characterized (49–53), alkane degraders belonging to the *Myxococcota* and *Planctomycetota* phyla are poorly studied. Our phylogenetic analysis coupled with the identification of alkane oxygenase genes in taxonomically distant MAGs indicates that there might be a relatively high diversity of unidentified alkane degraders in the biofilm of PE in the sea. The few studies that looked into the genetic potential for PE degradation in the ocean mainly focused on the *alkB* gene (28). After 2 years of incubation, the high relative abundance of MAGs with other non-AlkB alkane monooxygenases indicates that pathways other than those associated with *alkB* might also be relevant for the utilization of PE-originating compounds and should be considered in future research.

Even after 2 years of incubation, some of the MAGs apparently do not have the genes required for alkane oxidation or transport into the cell. This suggests that there might be other nonidentified pathways involved in the utilization of LDPE-derived carbon. This could, for example, explain the increase in relative abundance of several *Planctomycetota* MAGs in which no alkane oxidase was found. While two of the *Planctomycetota* MAGs had genes classified as alkane oxygenase genes, the majority did not. Albeit in low relative abundances, members of this phylum have been previously found in oil-degrading consortiums (54), and in fact, some have been reported to have alkane monooxygenase genes (55). As common elements of plastic biofilms (8, 56), the potential for members of this phylum to utilize PE-originating compounds should be considered.

The lack of genes in some MAGs related to alkane degradation, however, can also be a result of the low completeness of some of these genomes, not allowing the detection of the specific genes. It might also simply reflect the fact that some of these MAGs indeed do not have the ability to degrade hydrocarbons derived from LDPE and are sustained by by-products of the hydrocarbon-degrading component of the community or other compounds potentially leaching from the plastic or generated by the biofilm-associated microbes. Community B1 appears to also have been dominated by bacteria from groups R and K and MAG 5 (Fig. 4), giving the community the necessary repertoire of genes for surfactant production and alkane degradation. However, this community showed slower biofilm development than the other two communities despite exhibiting similar diversity (Fig. 6 and Fig. S1). Furthermore, nonweathered LDPE sheets lost less weight when incubated with community B than with either of the other two communities (Fig. 10). This might be a result of differences in LDPE degradation efficiency between the different organisms in each of the incubations but might also suggest that community B might have been limited in specific compounds that communities A and C were able to produce. This implies that if the degradation of LDPE-related hydrocarbons does in fact take place, the degraders, while capable of sustaining growth of a diverse bacterial community, might be relatively slow growing and require metabolic diversity to efficiently degrade LDPE-associated compounds. This is in accordance with laboratory experiments on the degradation of untreated PE by bacterial monocultures, exhibiting low biodegradation rates (21, 57). Generally, the FTIR spectra revealed that changes in the surface of the PE incubated with the three communities were relatively small after 6 months. However, bacterially mediated modifications of the surface of the plastics, especially on nonweathered LDPE by community A and on packaging PE when incubated with community C, caused an apparent weight loss of these plastics after 8 months of incubation (Fig. 10). Furthermore, holes and cracks appeared in some of the plastics incubated with bacteria (Fig. 9C, G, and H). This might indicate that the communities degraded the surface of the plastic at very low rates, taking up the oxidation products at the same rate as they were set free from the LDPE. Thus, most changes in the surface structure were small or undetectable by our ATR-FTIR analysis (Fig. 7). It might also indicate that the communities mainly utilized compounds passively released by the plastics into the surrounding seawater, rather than actively degrading the polymer. Previously, it has been reported that the same LDPE sheets as used in this study released dissolved organic carbon (DOC) into the surrounding seawater, even when kept in the dark. This released DOC could subsequently be taken up by the marine microbial community (58). In our study, it was not possible to determine whether the bacterial consortiums persisted by utilizing the LDPE polymer or the LDPE-associated additives, potentially leaching into the environment (59). Recent evidence shows that certain bacteria can degrade specific plastic additives, some through pathways that also lead to fatty acid beta-oxidation (60). Utilization of additives, rather than the more stable LDPE polymeric structure, could also explain the decrease in weight of the LDPE pieces and cracking of the plastic surface, while also explaining the low signs of surface oxidation shown by the ATR-FTIR analysis. The degradation of these compounds in the marine environment is only poorly studied, and to date, there is no evidence that alkane degraders like the ones enriched in our incubations can also degrade additives. However, due to the lack of knowledge of pathways involved in the biodegradation of most additives and the high variety of additive compounds, the possibility that certain organisms in our communities can utilize leaching LDPE additives as energy and carbon sources should not be disregarded.

There was also evidence of abiotic oxidation of plastics, as shown by low oxygen consumption in the controls (where microbes were absent) and in the ATR-FTIR spectra of the previously UV-weathered plastics. In our experiments, abiotic oxidation of plastics was most obvious in the incubations with UV-weathered LDPE sheets (Fig. 7), where chemical compound classes were not detected in nonweathered LDPE and carbonyl groups decreased after 6 months of incubation even in the abiotic control (61). Interestingly, the CI was significantly higher in UV-treated LDPE incubated with community C than in the pieces without microbial communities (Fig. 8), which suggests that community C might be the most efficient consortium in utilizing LDPE-derived compounds. This difference in CI values between the control samples and samples incubated with the microbial communities was not observed either in the nontreated LDPE or in the packaging incubations. This supports the idea that plastic biodegradation by microorganisms is more efficient when the polymer has previously undergone partial photooxidation (62). However, although relatively higher CI median values were observed on UV-treated LDPE incubated with communities A and B than on the controls, the differences were statistically not significant (Table 1). This reinforces the fact that if LDPE biodegradation occurs, it takes place at relatively low rates, even when the plastic was previously UV exposed.

Several heterotrophic bacteria like the organisms from group R (Fig. 4) (*Rhodobacteraceae*) might have the ability to use alkanes as carbon and energy sources. Whether they utilize plastics in the presence of other carbon sources, however, remains unknown. *Rhodobacteraceae* are typically among the most abundant families found in marine biofilms and are commonly enriched in plastic biofilms (63–66). Although the group R MAGs were slightly enriched over time, they were already relatively abundant in the original community A0. They all harbored a gene encoding at least one alkane monooxygenase, and the gene *fadL*, encoding a fatty acid transporter, was detected in all except one. In natural marine biofilms, these organisms might have access to more easily accessible and metabolizable carbon and energy sources and therefore might not participate in the uptake of LDPE-derived hydrocarbons. In contrast, obligatory hydrocarbonoclastic organisms, like Alcanivorax borkumensis, are specialized and dependent on degrading hydrocarbons (31). Group K organisms (Fig. 4) did exhibit several metabolic similarities and were closely related to Alcanivorax borkumensis, potentially occupying the same or a similar metabolic niche in the environment. For example, MAG 56, one of the most enriched MAGs, appears to lack proteins involved in the transport of sugars and amino acids into the cell. This might be an indication that similar to Alcanivorax borkumensis, this bacterium relies exclusively on alkanes as a carbon source. However, we did not obtain completely closed genomes for any of the organisms in our incubations, which makes it impossible to determine with certainty whether they really lack these functions. Interestingly, even if present in relatively low abundances, the family *Alcanivoraceae* has been associated with plastics, including PE, in the marine environment (4, 67, 68). This might be an indication that these bacteria utilize PE as a carbon and energy source in the environment, consequently playing a role in the fate of plastics in the sea.

The lack of metagenomic data for the original communities B and C does not allow us to statistically determine MAGs and genes that were enriched after 1 year of incubation, when the major shifts in the community composition might have occurred. However, the development of communities A and C into relatively similar communities over time and the increase in abundance of taxa related to alkane and fatty acid degradation do support the hypothesis that alkane-degrading pathways are related to the degradation of PE-associated compounds. Taken together, our results show that we have identified a plethora of organisms, pathways, and genes, other than the gene *alkB*, which might play an important role in the utilization of LDPE-associated compounds in the marine environment, thus providing a strong starting point for future studies addressing the mechanisms underlying microbial biodegradation of LDPE in the ocean.

## MATERIALS AND METHODS

### Experimental setup.

We collected three plastic pieces from the sea surface of the Northern Adriatic Sea, approximately 500 m off the coast of Rovinj, Croatia, at around 45°05′29.1″N 13°37′44.5″E. Two were collected in November 2016 (plastics A and B) and one was collected in August 2016 (plastic C). The plastics were immediately taken to the laboratory submerged in seawater (Fig. S5). A piece from each plastic was cut and frozen at −80°C for DNA analysis and another piece was taken for Raman spectroscopy to determine the plastic type. The three plastics were classified as polyethylene (PE). The remaining plastics were transferred to separate 1-L borosilicate bottles filled with artificial seawater (ASW). The ASW composition was as follows: 355.2 mM NaCl, 24.48 mM Na_2_SO_4_, 7.75 mM KCl, 2.02 mM NaHCO_3_, 0.71 mM KBr, 0.36 mM H_3_BO_3_, 0.06 mM NaF, 46.21 mM MgCl_2_·6H_2_O, 8.95 mM CaCl_2_·2H_2_O, 0.08 mM SrCl_2_·6H_2_O, 0.01 mM NH_4_Cl, and 0.002 mM NaH_2_PO_4_. The bottles were kept in the dark at room temperature (21°C to 22.5°C).

10.1128/msystems.01415-21.7 FIG S5 Plastics A, B, and C. Download FIG S5, TIF file, 2.7 MB. Copyright © 2022 Pinto et al. 2022 Pinto et al. https://creativecommons.org/licenses/by/4.0/ This content is distributed under the terms of the Creative Commons Attribution 4.0 International license.

After 3 weeks, the plastics were transferred to fresh 1-L bottles with ASW. This process was repeated seven times for plastic C and four times for plastics A and B. One small piece of each plastic type was then cut and incubated in a new bottle with ASW amended with inorganic nutrients (as described above) together with sterile low-density polyethylene (LDPE) pieces. The LDPE sheets were colorless, 0.5 mm thick, cut from an LDPE film purchased from Goodfellow (product code ET31-FM-000352 as of 2022). Regarding additives, we were informed by Goodfellow that this plastic did not contain fire retardants or plasticizers but could have contained antioxidants used during production. Additionally, in a previous study (8), we used the same LDPE for another experiment, and it did not contain measurable phthalate concentrations. Each bottle contained 10 LDPE squares (1 by 1 cm). After 3 weeks, one of these LDPE pieces was transferred to a new bottle with ASW, again together with new sterile LDPE pieces. This process was repeated for 2 years. After 1 and 2 years, one of the above-mentioned originally sterile LDPE plastics, from each of the incubations, was taken and frozen at −80°C for metagenomics analyses. After 2 years, samples were also taken for respiration measurements. All bottles were kept in the dark at room temperature and manually shaken once a day.

All tools and bottles used were made of materials other than plastic and were all sterilized. All glass and metal objects were sterilized in a muffle furnace at 500°C for 30 min.

### DNA extraction and sequencing.

DNA from plastics was extracted using a modified bead-beating approach combined with the Puregene tissue DNA extraction kit (Qiagen, Valencia, CA) as previously described (69). Samples with a sufficient amount of DNA were single-end sequenced (1 × 150 bp) with Illumina NextSeq technology at Microsynth AG, Switzerland. These were plastic A right after collection (A0), after 1 year of incubation (A1), and after 2 years of incubation (A2), plastic C after 1 year and 2 years of incubation (C1 and C2, respectively), and plastic B after 1 year of incubation (B1). All raw sequences files are available from the NCBI Sequence Read Archive (SRA) database (BioProject PRJNA599974). Due to low DNA concentrations, samples from plastics B and C right after collection (samples B0 and C0) and sample B2 were not sequenced.

### Oxygen consumption measurements.

At the end of the incubations after 2 years, one plastic piece from each of the incubations was transferred to a new bottle with ASW together with 16 sterile LDPE pieces. A second plastic piece from each of the incubations was incubated in a separate bottle with ASW with 16 glass pieces of 1 cm by 2.25 cm. As a control, 16 sterile LDPE pieces with the dimensions 1.5 cm by 1.5 cm were incubated in a separate bottle with ASW (control I). In order to let the biofilm develop on both glass and plastic without the influence of the old LDPE pieces, after 1 month, the plastics and glass were transferred to new ASW bottles and incubated for 2 additional months.

After 2 months, 15 pieces from each of the incubations were distributed among three BOD (biological oxygen demand) bottles of approximately 120 mL filled with ASW, resulting in triplicate bottles with five plastic pieces each. The same was done for the glass samples. One piece was also taken for bacterial abundance determination by adding it to 4% formaldehyde for 10 min and storing it at −80°C. Before transferring the glass and plastic to the BOD bottles for oxygen consumption measurements, one piece of glass and one of LDPE from each of the incubations were taken to determine cell abundance.

All the BOD flasks were kept at 21°C for 24 h prior to the experiment. Additionally, before adding the plastic pieces to the BOD bottles, they were put in petri dishes filled with the same ASW and slightly bent to fit them through the narrow end into the BOD bottles. The bottles were filled up to half with ASW, and then the plastics were carefully placed inside and the rest of the bottle was gently filled with ASW. Care was taken to avoid any air bubbles enclosed in the BOD flasks. Additionally, three control bottles were added by incubating sterile LDPE pieces that had not been previously incubated in ASW (control II). The bottles were immediately submerged in a water bath kept at 21°C and incubated for approximately 40 h. For determining the initial oxygen concentration in the ASW (T0), three BOD bottles filled only with ASW were fixed immediately and thoroughly shaken.

The respiration measurements followed the protocol described by Labasque et al. (70) for spectrophotometric Winkler determination of dissolved oxygen. After fixation and addition of sulfuric acid, absorbance was measured using a Shimadzu UV-visible (UV-Vis) 1800 PC spectrophotometer with a flowthrough cell connected to a Masterflex L/S peristaltic pump within 5 min. Flow was maintained at around 10 mL min^−1^.

Measurements were done in a temperature-controlled room (set to 21°C). The amount of total iodine and tri-iodide in the samples was determined at a wavelength of 466 nm. The spectrophotometer was calibrated using standard additions of potassium iodate to BOD bottles filled with ASW to which the Winkler chemicals were added in reverse order.

### Cell abundance.

The samples were washed in sterile ASW, immersed in 2% formaldehyde, and after 10 min stored at −80°C. A 4′,6-diamidino-2-phenylindole (DAPI; 2-μg mL^−1^ final concentration) mix was used to stain the cells attached to the surface of one of the sides of the glass and plastic pieces. Each sample was placed on a drop of Milli-Q water on a glass slide, a drop of DAPI mix was pipetted onto the entire exposed surface of the sample, and a coverslip was placed on top of the sample. After 10 min of incubation in the dark, the samples were analyzed under an epifluorescence microscope (Axio Imager M2; Carl Zeiss; magnification, ×1,250). Between 35 and 50 photos were taken from different randomly selected locations of each of the samples, corresponding to averages of 1.95 × 10^7^ ± 6.17 × 10^6^, 1.22 × 10^7^ ± 3.67 × 10^6^, and 4.05 × 10^7^ ± 1.52 × 10^7^ cells on plastics incubated with communities A, B, and C, respectively, and averages of 5.68 × 10^6^ ± 3.02 × 10^5^, 3.14 × 10^5^ ± 1.63 × 10^5^, and 2.88 × 10^5^ ± 2.03 × 10^5^ cells on the glass pieces incubated with communities A, B, and C, respectively (Fig. S2).

The number of DAPI-stained cells per photo was counted with the program ImageJ2 (71). To determine the number of cells per square centimeter on each surface, the number of cells on each photo was divided by the area of one photo (1.4 × 10^−4^ cm^2^). Subsequently, the total number of cells in each of the incubations was estimated by multiplying the number cells per square centimeter on each photo by the total area of the plastics and glass (4.5 cm^2^) and averaging the result from all photos per treatment.

### Assessing the physical and chemical properties of the PE surface.

To determine changes of the surface of PE sheets when incubated with the three communities after 2 years, we incubated one plastic colonized with one of the three communities in sterile ASW with three different types of PE pieces: (i) the same LDPE sheets as mentioned above, (ii) the same LDPE sheets but previously UV-weathered (irradiated with UV radiation; wavelength, 300 to 400 nm) under artificial conditions in a SUNSET CPS+ (Atlas-MTS) for 2 weeks, and (iii) PE sheets from a pear packaging bag bought in a supermarket. Twenty pieces of each of the different types of PE sheets were incubated in 500-mL bottles, filled to 300 mL with ASW and with one plastic already colonized with one of the three communities. A control bottle for each of the PE sheet types was prepared without the addition of an already colonized plastic. The bottles were kept at 21.5°C. After 3 months of incubation, one plastic sample from each bottle was collected for scanning electron microscopy (SEM) and at least quadruplicate samples were collected after 6 months for attenuated total reflection Fourier-transform infrared (ATR-FTIR) analysis. After 8 months of incubation, at least five plastics were weighed from each incubation.

### ATR-FTIR analysis.

To characterize changes of the surface of the plastic material and avoid interference of the biofilm itself in the ATR-FTIR analysis and plastic weight, the biofilm was removed prior to the ATR-FTIR analysis and the weighing of the plastic. To remove the biofilm, the plastic pieces were immersed in 2% SDS in a Greiner tube; the tube was briefly vortexed and then left at room temperature for 4 h. Thereafter, the plastics were immersed in Milli-Q water in a new Greiner tube, the tube was vortexed again, and then the plastics were carefully dried and wrapped in clean paper, and kept at 4°C until analysis. Prior to ATR-FTIR analysis, the plastics were again dried with clean paper.

ATR-FTIR was used to detect the functional chemical groups of the PE sheets. A Bruker Tensor 27 FTIR spectrometer (glowbar mid-infrared spectroscopy [MIR] light source, KBr beam splitter, DLaTGS detector) equipped with a Harrick MVP2 diamond ATR unit was used and the spectra were obtained and processed with the Bruker program OPUS 5.5. Spectra were acquired from 4,000 to 370 cm^−1^ with 4 cm^−1^ resolution. To obtain a good signal-to-noise ratio, spectra were averaged from each 32 scans. Background scans from the empty ATR unit were performed prior to sample analysis. At least three separate plastic pieces of each of the incubations were analyzed. In order to evaluate the presence of oxidation of each of the plastics in each treatment, the carbonyl index (CI) was calculated by using the specified area under band (SAUB) method described by Almond et al. (72). The CI was calculated by dividing the integrated area under band 1,850 to 1,650 cm^−1^ with the area under band 1,500 to 1,420 cm^−1^. The areas under bands were integrated for each sample for each replicate.

To determine whether the bacterial communities influenced plastic weight, the pieces were weighed on a Mettler MT5 balance. A hole puncher was used to obtain pieces of the exact same size (0.5-cm diameter) of the nonweathered LDPE. For the packaging PE, the originally incubated 1 cm^2^ pieces were weighed. Due to extremely irregular shapes of the UV-weathered pieces and their brittleness, which did not allow for further manipulation, they were not considered for this part of the experiment.

### SEM analysis.

SEM analyses were performed on plastics after removing the biofilm and without removing the biofilm. Samples for SEM were incubated in 2% glutaraldehyde for 10 min and then stored at −80°C until further processing. SEM samples were dehydrated with an ethanol dilution series of 30%, 50%, 70%, 80%, 90%, and 95% each for 10 min and three times in 100% absolute ethanol for 10 min. The dehydrated samples were CO_2_ critical-point dried with a CPD 300 auto-critical-point dryer (Leica Microsystems). The dried pieces were gold coated using a JFC-2300HR sputter coater (JEOL Ltd.) for 80 s. Pictures were taken using a secondary electron detector with a JEOL JSM-IT300 scanning electron microscope with 15-kV acceleration voltage in ultrahigh vacuum.

### Genomic assembly and annotation.

Read quality was assessed with Fastqc (73). Overrepresented sequences were removed and reads were shortened to 149 bp in all samples using the AdapterRemoval program (74). After filtering and trimming, totals of 35,326,547, 15,541,923, 38,372,532, 3,180,678, 35,388,001, and 37,988,480 reads from samples A0, A1, A2, B1, C1, and C2, respectively, were coassembled using the program Megahit (75) with default settings. Since we were aiming at identifying organisms and pathways enriched in the cultures, sample A0 was excluded from the coassembly to decrease complexity.

Predicted genes were identified from the coassembly using the Prodigal (76) software with default settings. Functional annotation of the predicted genes was performed with the online EggNOG-mapper v1 (77) using Diamond as the sequence aligner with a maximum E value cutoff of 10^−5^. A total of 2,500,984 predicted genes were recovered from the coassembly, from which 736,382 were annotated with the EggNOG emapper and from which 9,532 unique KO orthologies were identified. Over 74% of the genes were assigned to a COG category. A total of 958 genes increased in RPKM (reads per kilobase million) value over time (Table S2), of which 154 were classified as oxidoreductase genes (Table S2).

Kaiju (78) was used for the taxonomic classification of the metagenome reads using the NCBI BLAST *nr*+euk database directly on the Kaiju server. For a more precise classification of the prokaryotic communities, rRNA genes from each individual sample were assembled and further annotated through matam (79) using Silva_132_SSURef_Nr99 as the reference database. The identification of OTUs with matam is directly dependent on the correct assembly of one single gene (16S rRNA gene). Hence, it is likely to underestimate diversity in samples with a low number of reads by failing to assemble the gene of rare species. Metagenomes A1 and B1 had a significantly lower number of reads than the other samples. Therefore, to compare OTU diversity among all samples, the relative abundance and counts of OTUs were determined by the software mOTUs (80). This software classifies OTUs based on a group of 40 universal marker genes, and hence, it is likely to be less biased toward identifying only relatively abundant OTUs than matam.

OTU diversity indexes Shannon and Simpson and OTU richness were calculated using the iNext R package (81). Coverage-based rarefaction was used to normalize all samples to a coverage of 84%, corresponding to the calculated coverage of the B1 metagenome, which was the lowest of all samples. The bioinformatic pipeline used in this study can be found in Text S2.

10.1128/msystems.01415-21.2 TEXT S2 Bioinformatics pipeline. Download Text S2, TXT file, 0.05 MB. Copyright © 2022 Pinto et al. 2022 Pinto et al. https://creativecommons.org/licenses/by/4.0/ This content is distributed under the terms of the Creative Commons Attribution 4.0 International license.

### Statistical analysis and bioinformatics.

Analysis of variance (ANOVA) was performed to compare differences in total oxygen consumption and cell-specific oxygen consumption rates between samples with different communities and substrates. To assess differences between specific pairs of samples, *post hoc* Tukey tests were performed. An ANOVA and *post hoc* Tukey tests were also performed to compare the weights of plastics incubated with different communities after 8 months of incubation. To identify genes enriched after 2 years of incubation in communities A and C, in comparison to the same communities incubated after 1 year, a differential-expression analysis was performed using the DESeq2 (82) analysis tools in R (83). Gene counts previously obtained with bbmap were used as input for the DESeq2 analysis. KEGG (84)-annotated genes with significant differences in their fold change in abundance, determined by the DESeq2 analysis (*P* value < 0.05), between the two years were compared to the selected genes potentially involved in alkane and fatty acid biodegradation (Text S1). Communities B and C were used as replicates, with incubation time used as a variable. All statistical analyses and plots were done with the program R, unless otherwise stated.

### Read mapping.

To obtain coverage information for assembled contigs and bins in all samples, the reads were mapped to the assembled contigs using the Burrows-Wheeler Aligner (BWA) (85). The mapped read counts, coverage, and RPKM were extracted using bbmap/pileup (86).

### Binning.

To recover individual genomes of the organisms enriched in the incubations, contigs from the coassembly with a minimum of 1,500 bp were binned using metabat (87) and maxbin (88). In both cases, both tetranucleotide frequency and contig coverage in every individual sample were used for binning.

To obtain high-quality draft genomes, the resulting bins from each program were refined using metaWRAP (89). The resulting refined bins with completeness of >50% and contamination of <10% were used for further analysis. Here, bins are called metagenome-assembled genomes (MAGs).

Taxonomic classification of the MAGs was conducted via phylogenetic inference through marker genes using the software GTDB-Tk, which uses the taxonomy as suggested by the Genome Taxonomy Database (42). We used Fasttree (90) to build a phylogenetic tree with the multiple-sequence alignment output by GTDB-Tk (91). MAG coverage in each sample was calculated by dividing the total number of base pairs of reads mapped to the MAG by the total length of all contigs and multiplying it by 10^6^ divided by the total number of base pairs of the sample. The count of mapped reads to the contigs of each MAG was obtained as described above. Gene calling of each MAG was predicted using Prodigal. Sequences encoding AlkB and cytochrome P450 were retrieved by HMMER search (92) (hmmscan) against a curated model downloaded from FunGene (http://fungene.cme.msu.edu/) with a threshold of domain coverage of >30%. The gene encoding CYP153 was further refined from the cytochrome P450 gene with a customized HMM with a domain coverage of >75%. MAFFT (93) was used to build the alignment for both the *alkB* and CYP153 genes. The phylogenetic tree was generated using RAxML (94) under the PROTGAMMALG model with 100 bootstraps. Tree visualization was carried using the Interactive Tree Of Life (iTOL) webtool (95).

### Enriched genes and metabolic pathways.

Because oxidoreductase profiles have been found to be best suited to functionally characterize a microbial community (96), in addition to determining genes that increased in RPKM value over time, we specifically looked at the oxidoreductases (the studied oxidoreductases can be found in Table S2). Oxidoreductases were obtained by selecting KEGG orthology (KO) annotations obtained with EggNOG and classified as oxidoreductases (EC 1). The EC numbers were obtained using KO to EC information provided by the ko2ec tool. The KO numbers of enriched genes (genes can be found in Table S2) were used as an input into the online KEGG Mapper reconstruction option, and the complete enriched pathway modules were identified. We also analyzed the relative abundances of selected genes (Text S1) encoding enzymes previously suggested in the literature as being related to alkane degradation in all our metagenomes and determined their presence in the individual MAGs.

### Data availability.

All raw sequences files are available from the NCBI SRA database (BioProject: PRJNA599974).
