# Supplementary figures and images for: Microbial Consortiums of Putative Degraders of Low-Density Polyethylene-Associated Compounds in the Ocean
Source: mSystems. 2022 Mar 1;7(2):e01415-21. doi: 10.1128/msystems.01415-21 (PMC8941889; doi:10.1128/msystems.01415-21)

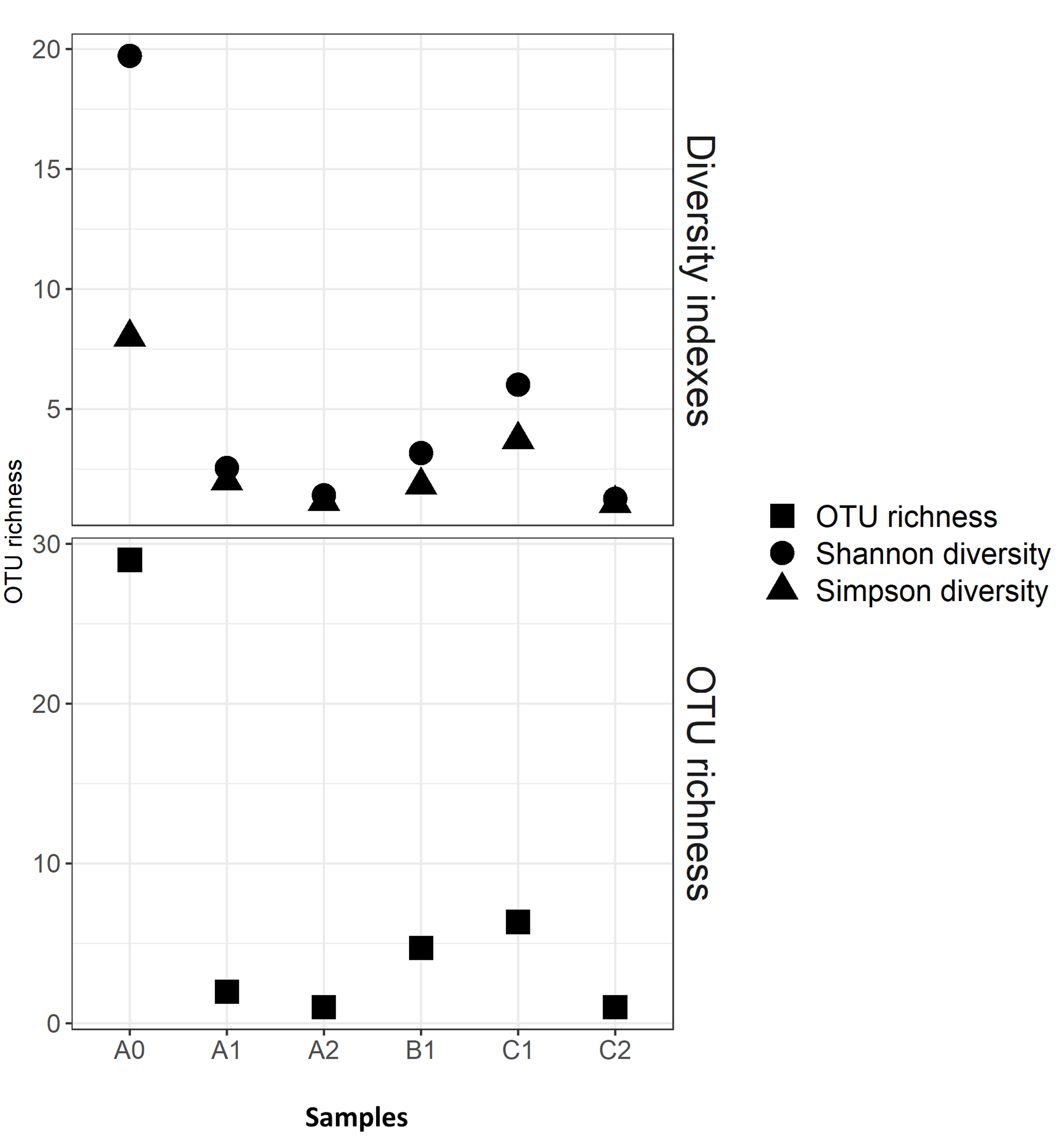

Supplement: FIG S1 [file msystems.01415-21-sf001.tif]

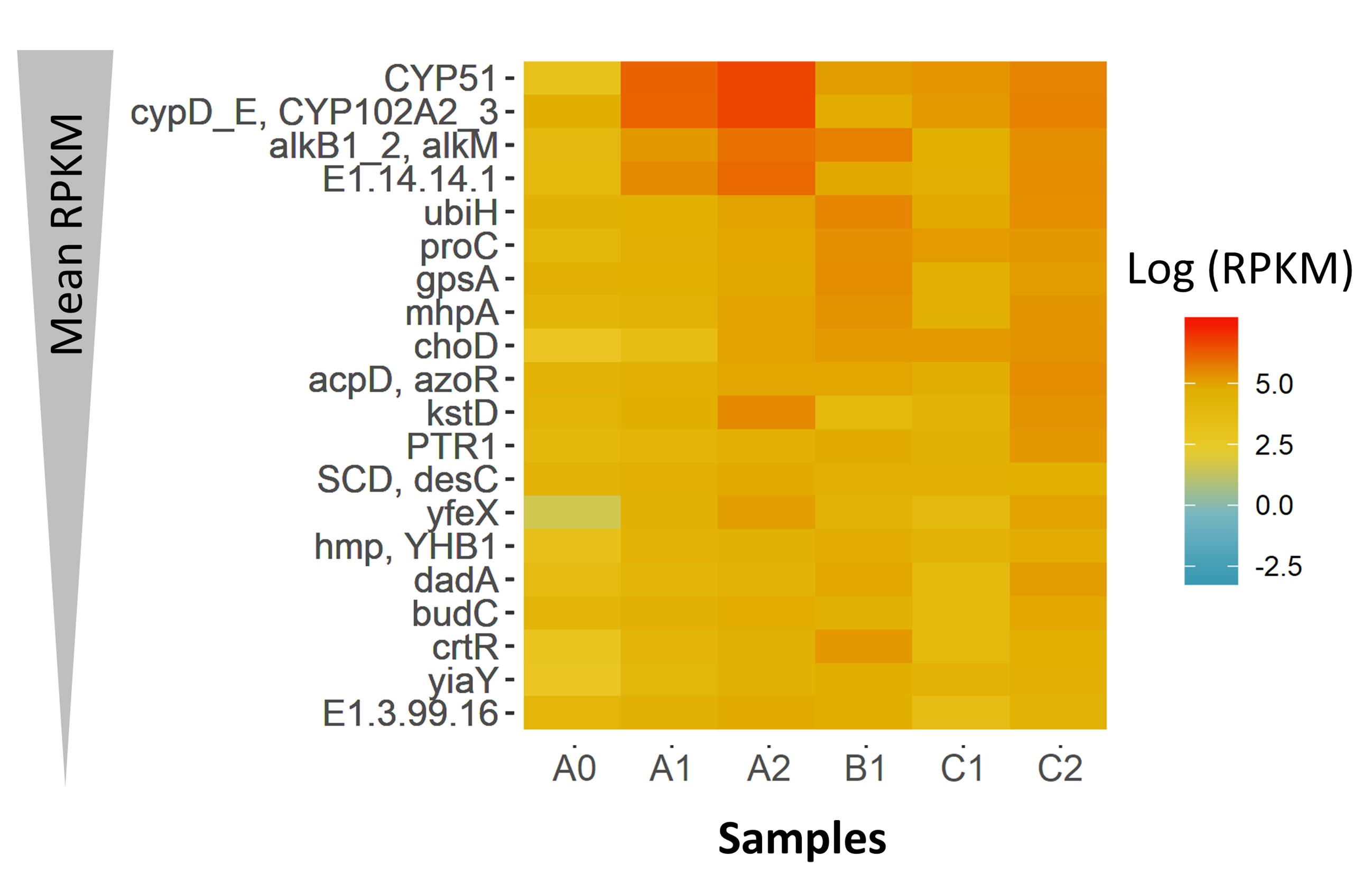

Supplement: FIG S2 [file msystems.01415-21-sf002.tif]

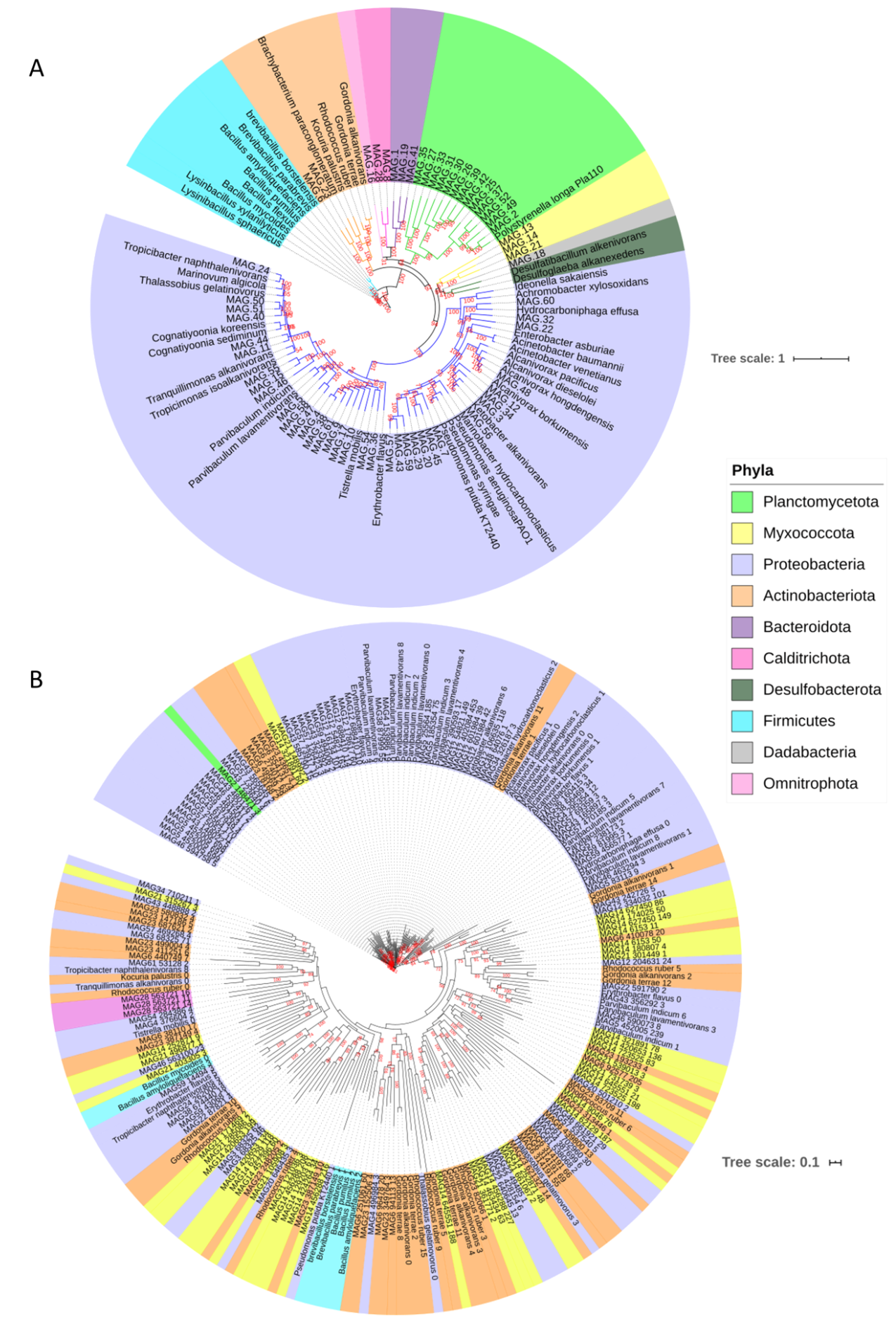

Supplement: FIG S3 [file msystems.01415-21-sf003.tif]

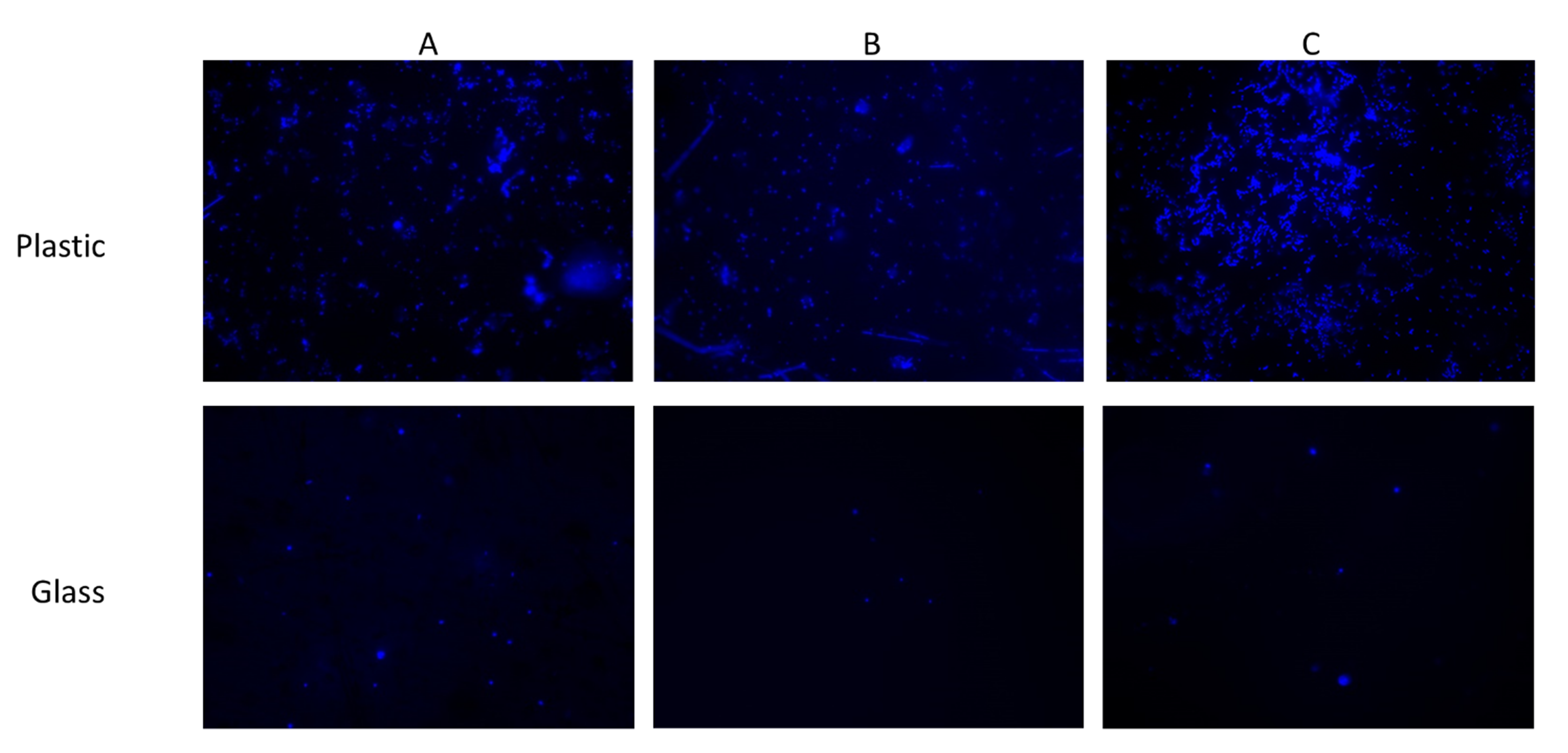

Supplement: FIG S4 [file msystems.01415-21-sf004.tif]

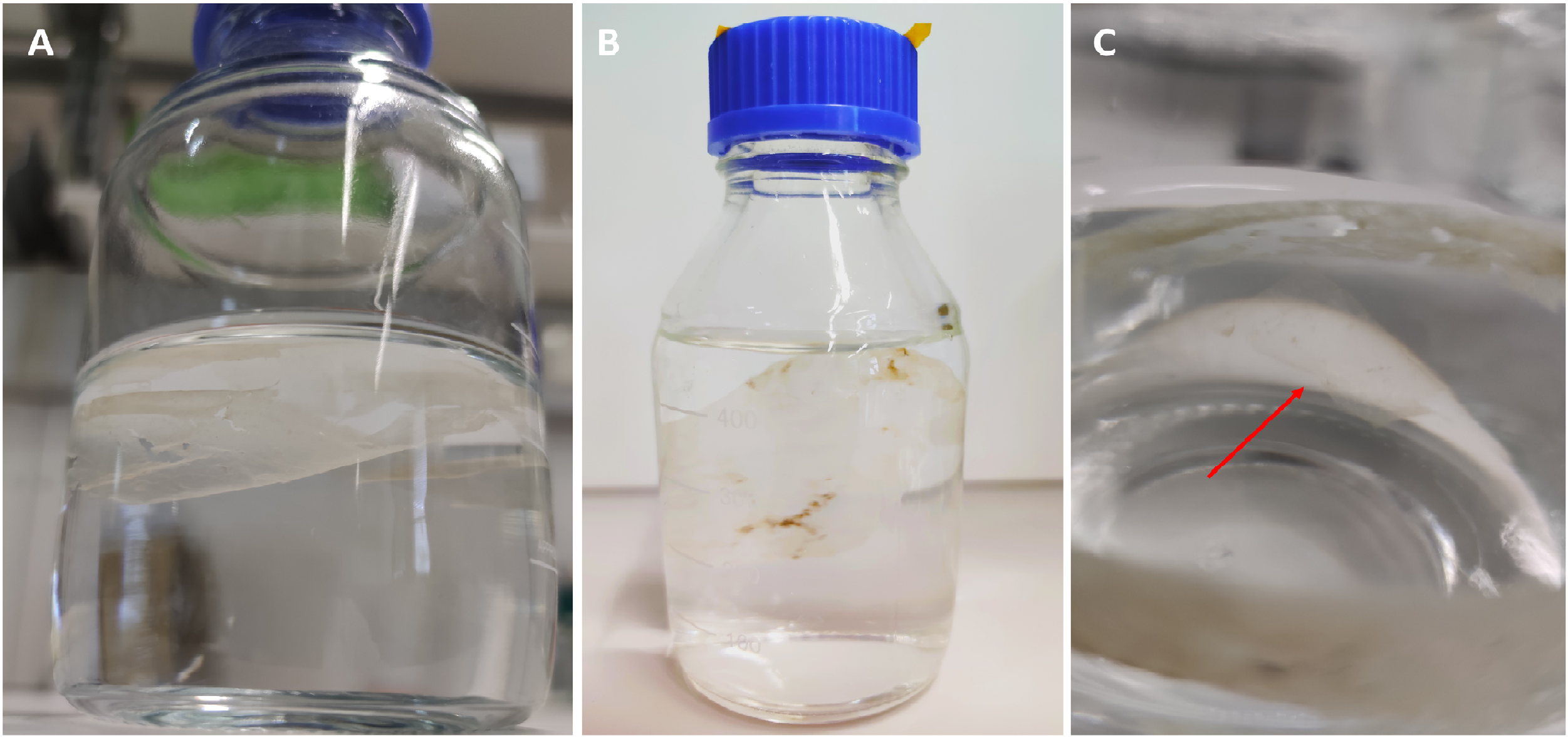

Supplement: FIG S5 [file msystems.01415-21-sf005.tif]
