# Supplementary material for: Microbial Consortiums of Putative Degraders of Low-Density Polyethylene-Associated Compounds in the Ocean
Source: mSystems. 2022 Mar 1;7(2):e01415-21. doi: 10.1128/msystems.01415-21 (PMC8941889; doi:10.1128/msystems.01415-21)
Supplement: TEXT S1 [file msystems.01415-21-s0001.pdf]

Selected genes encoding for enzymes previously suggested to be involved in alkane and fatty acid biodegradation. Genes in grey were not found in any of MAGs. *This gene has not been attributed a KO identification.

KEGG number	Gene name	Function	Cluster
K00496	alkB1_2, alkM	Alkane 1-monooxygenase	(A) AlkB cluster
K05297	rubB, alkT	Rubredoxin---NAD+ reductase	
K21738	alkT	Rubredoxin---NAD+ reductase	
K22567	nroR	Rubredoxin---NAD+ reductase	
NA*	CYP153	Cytochrome P450 alkane hydroxylase	(B) CYP153 cluster
K04755	fdx	ferredoxin, 2Fe-2S	
K05524	fdxA	ferredoxin	
K21567	fnr	ferredoxin/flavodoxin---NADP+ reductase	
K00528	fpr	ferredoxin/flavodoxin---NADP+ reductase	
K18914	fdxR	adrenodoxin-NADP+ reductase	
K00529	hcaD	3-phenylpropionate/trans-cinnamate dioxygenase ferredoxin reductase component	
K18223	prmA	propane 2-monooxygenase large subunit	(C) Propane oxygenase cluster
K18224	prmC	propane 2-monooxygenase small subunit	
K18225	prmB	propane monooxygenase reductase component	
K18226	prmD	propane monooxygenase coupling protein	
K10944	pmoA-amoA	Methane/ammonia monooxygenase subunit A	(D) Methane oxygenase cluster
K10945	pmoB-amoB	Methane/ammonia monooxygenase subunit B	
K10946	pmoC-amoC	Methane/ammonia monooxygenase subunit C	
K20938	LadA	Long-chain alkane monooxygenase	(E) Long-chain alkane related monooxygenases and fatty acid oxygenases
K00485	AlmA	Dimethylaniline monooxygenase/flavin containing monooxygenase	
K07425	CYP4_A	long-chain fatty acid omega-monooxygenase	
K15401	CYP86A1	long-chain fatty acid omega-monooxygenase	
K20495	CYP704B1	long-chain fatty acid omega-monooxygenase	
K22887	CYP52M1	long-chain fatty acid omega-monooxygenase	
K00493	E1.14.14.1	Unspecific monooxygenase [EC:1.14.14.1]	
K06076	fadL	Long-chain fatty acid transport protein	(F) Transporters and biosurfactant synthesis
K10940	OmpT	Outer membrane protein/ long-chain fatty acid transport protein involved in the alkane assimilation	
K07275	OmpW, alkL	Outer membrane protein (surfactant and transports 1-alkanes into the cell)	
K06142	OmpH	Outer membrane protein (surfactant)	
K18100	rhlA	Rhamnosyltransferase subunit A (rhamnosyltransferase involved in rhamnolipid biosurfactant synthesis)	
K18101	rhlB	Rhamnosyltransferase subunit B (rhamnosyltransferase involved in rhamnolipid biosurfactant synthesis)	

K13954	yiaY	Alcohol dehydrogenase	(G) Alcohol dehydrogenases
K19954	adh1	Alcohol dehydrogenase	
K13951	ADH1_7	Alcohol dehydrogenase 1/7	
K13980	ADH4	Alcohol dehydrogenase 4	
K13952	ADH6	Alcohol dehydrogenase 6	
K13953	adhP	alcohol dehydrogenase, propanol-preferring	
K00001	adh	Alcohol dehydrogenase	
K00121	frmA, ADH5, adhC	S-(hydroxymethyl)glutathione dehydrogenase / alcohol dehydrogenase	
K04072	adhE	Acetaldehyde dehydrogenase / alcohol dehydrogenase	
K18857	ADH1	alcohol dehydrogenase class-P	
K00128	ALDH	Aldehyde dehydrogenase (NAD+)	(H) Aldehyde dehydrogenases
K14085	ALDH7A1	Aldehyde dehydrogenase family 7 member A1	
K00149	ALDH9A1	Aldehyde dehydrogenase family 9 member A1	
K21747	alkR	AraC family transcriptional regulator, alkane utilization regulator	(I) Transcriptional regulators
K21748	alkS	LuxR family transcriptional regulator, alkane degradation pathway regulator	
K00375	gntR	GntR family transcriptional regulator / MocR family aminotransferase	
K08365	merR	MerR family transcriptional regulator, mercuric resistance operon regulatory protein	
K18476	tetR	TetR/AcrR family transcriptional regulator, tetracycline repressor protein	
K02099	araC	AraC family transcriptional regulator, arabinose operon regulatory protein	

List of bacterial strains associated with hydrocarbons or plastics degradation.

Strain	Type of substrate	Additional information	Environment	Reference
Rhodococcus ruber	PE and PS		Culture	[1]
Brevibacillus borstelensis	PE		Soil	[2]
Bacillus mycoides	PE	Same as *Bacillus weihenstephanensis*	Soil	[3]
Bacillus amyloliquefaciens	PE		Guts of waxworms	[4]
Bacillus pumilus	PE		Seawater	[5]
Bacillus subtilis	PE		Seawater	[5,6]
Achromobacter xylosoxidans	PE		Soil	[7]
Enterobacter asburiae	PE		Guts of waxworms	[4]
Kocuria palustris	PE		Seawater	[5]
Lysinbacillus xylanilyticus	PE		Soil	[8]
Pseudomonas aeruginosa PAO1	PE		Culture	[9]
Pseudomonas putida KT2440	PE		Culture	[9]
Pseudomonas syringae	PE		Culture	[9]
Brevibacillus parabrevis	PE		Landfill	[10]
Acinetobacter baumannii	PE		Landfill	[10]
Lysinibacillus sphaericus	PE	Same as *Bacillus sphaericus*	Seawater	[11]
Ideonella sakaiensis	PET		Sediments	[12]
Desulfatibacillum alkenivorans	Alkanes		Oil-polluted sediments	[13]
Alcanivorax pacificus	Alkanes		Deep sea sediments	[14]
Acinetobacter venetianus	Alkanes		Venice Lagoon	[15]
Tistrella mobilis	Alkanes		Seawater	[16]
Gordonia terrae	Alkanes		Oil-contaminated soil	[17]
Brachybacterium paraconglomeratum	Alkanes	Surfactant-producing bacteria	Corn steep liquor	[18,19]
Erythrobacter flavus	Alkanes		Seawater	[19,20]
Bacillus flexus	Alkanes		Seawater	[19,21]
Parvibaculum indicum	Alkanes		Seawater	[22]
Tropicimonas isoalkanivorans	Alkanes		Seawater from port	[23]

Tranquillimonas alkanivorans	Alkanes		Seawater from port	[24]
Desulfoglaeba alkanexedens	Alkanes		Naval wastewater-storage facility	[25]
Gordonia alkanivorans	Alkanes		Tar-contaminated soil	[26]
Ketobacter alkanivorans	Alkanes		Seawater	[27]
Alcanivorax dieselolei	Alkanes		Seawater and deep-sea sediments	[28]
Alcanivorax hongdengensis	Alkanes		Seawater	[29]
Alcanivorax borkumensis	Alkanes	Surfactant-producing bacteria	Seawater	[30]
Hydrocarboniphaga effusa	Alkane and aromatic hydrocarbon		Oil-contaminated soil	[31]
Parvibaculum lavamentivorans	Initiates catabolism of linear alkylbenzenes ulfonate (surfactants)		Activated sludge	[32]
Tropicibacter naphthalenivorans	PAH		Seawater from a port	[33]
Thalassobius gelatinovorus	PAH		Seawater	[34]
Marinobacter hydrocarbonoclasticus	Hydrocarbons		Seawater	[35]
Polystyrenella longa Pla110	PS	Isolated from PS particles, but no indication that it can degrade PS	Seawater	[36]
Marinovum algicola	Oil	Previously *Ruegeria algicola*	Seawater	[37]
*Cognatiyoonia koreensis**	PE	Previously *Loktanella koreensis*	Sea sand	[38], [39]
*Cognatiyoonia sediminum**	PE	Previously *Loktanella sediminum*	Marine sediments	[38], [39]

* Members from the *Cognatiyoonia* genus are associated with PE biofilms across different oceanic regions (unpublished data)

References

1. Sivan, A., Szanto, M. & Pavlov, V. Biofilm development of the polyethylene-degrading bacterium Rhodococcus ruber. *Appl. Microbiol. Biotechnol.* **72**, 346–352 (2006).
2. Hadad, D., Geresh, S. & Sivan, A. Biodegradation of polyethylene by the thermophilic bacterium Brevibacillus borstelensis. *J. Appl. Microbiol.* **98**, 1093–1100 (2005).
3. Ingavale, R. R. & Raut, P. D. Comparative biodegradation studies of LDPE and HDPE using Bacillus weihenstephanensis isolated from garbage soil. *Nat. Environ. Pollut. Technol.* **17**, 649–655 (2018).
4. Yang, J., Yang, Y., Wu, W.-M., Zhao, J. & Jiang, L. Evidence of polyethylene biodegradation by bacterial strains from the guts of plastic-eating waxworms. *Environ. Sci. Technol.* **48**, 13776–13784 (2014).
5. Harshvardhan, K. & Jha, B. Biodegradation of low-density polyethylene by marine bacteria from pelagic waters, Arabian Sea, India. *Mar. Pollut. Bull.* **77**, 100–106 (2013).
6. Ibiene, A. A., Stanley, H. O. & Immanuel, O. M. Biodegradation of polyethylene by Bacillus sp. indigenous to the Niger delta mangrove swamp. *Niger. J. Biotechnol.* **26**, 68–78 (2013).
7. Kowalczyk, A., Chyc, M., Ryszka, P. & Latowski, D. Achromobacter xylosoxidans as a new microorganism strain colonizing high-density polyethylene as a key step to its biodegradation. *Environ. Sci. Pollut. Res.* **23**, 11349–11356 (2016).
8. Esmaeili, A., Pourbabaee, A. A., Alikhani, H. A., Shabani, F. & Esmaeili, E. Biodegradation of low-density polyethylene (LDPE) by mixed culture of Lysinibacillus xylanilyticus and Aspergillus niger in soil. *PLoS One* **8**, e71720 (2013).
9. Kyaw, B. M., Champakalakshmi, R., Sakharkar, M. K., Lim, C. S. & Sakharkar, K. R. Biodegradation of low density polythene (LDPE) by Pseudomonas species. *Indian J. Microbiol.* **52**, 411–419 (2012).
10. Pramila, R., Padmavathy, K., Ramesh, K. V. & Mahalakshmi, K. Brevibacillus parabrevis, Acinetobacter baumannii and Pseudomonas citronellolis-Potential

candidates for biodegradation of low density polyethylene (LDPE). *African J. Bacteriol. Res.* **4**, 9–14 (2012).

11. Artham, T. *et al.* Biofouling and stability of synthetic polymers in sea water. *Int. Biodeterior. Biodegradation* **63**, 884–890 (2009).

12. Yoshida, S. *et al.* A bacterium that degrades and assimilates poly(ethylene terephthalate). *Science (80-.).* **351**, 1196–1199 (2016).

13. Cravo-Laureau, C., Matheron, R., Joulian, C., Cayol, J.-L. & Hirschler-Réa, A. Desulfatibacillum alkenivorans sp. nov., a novel n-alkene-degrading, sulfate-reducing bacterium, and emended description of the genus Desulfatibacillum. *Int. J. Syst. Evol. Microbiol.* **54**, 1639–1642 (2004).

14. Lai, Q. *et al.* Alcanivorax pacificus sp. nov., isolated from a deep-sea pyrene-degrading consortium. *Int. J. Syst. Evol. Microbiol.* **61**, 1370–1374 (2011).

15. Di Cello, F., Pepi, M., Baldi, F. & Fani, R. Molecular characterization of an n-alkane-degrading bacterial community and identification of a new species, Acinetobacter venetianus. *Res. Microbiol.* **148**, 237–249 (1997).

16. Wang, W., Wang, L. & Shao, Z. Diversity and abundance of oil-degrading bacteria and alkane hydroxylase (alkB) genes in the subtropical seawater of Xiamen Island. *Microb. Ecol.* **60**, 429–439 (2010).

17. Nicdao, M. A. C. & Rivera, W. L. Two strains of Gordonia terrae isolated from used engine oil-contaminated soil utilize short-to long-chain n-alkanes. *Philipp. Sci. Lett.* **5**, 1–10 (2012).

18. Kiran, G. S., Sabarathnam, B., Thajuddin, N. & Selvin, J. Production of glycolipid biosurfactant from sponge-associated marine actinobacterium Brachybacterium paraconglomeratum MSA21. *J. Surfactants Deterg.* **17**, 531–542 (2014).

19. Wang, L., Wang, W., Lai, Q. & Shao, Z. Gene diversity of CYP153A and AlkB alkane hydroxylases in oil-degrading bacteria isolated from the Atlantic Ocean. *Environ. Microbiol.* **12**, 1230–1242 (2010).

20. Harwati, T. U., Kasai, Y., Kodama, Y., Susilaningsih, D. & Watanabe, K. Characterization of diverse hydrocarbon-degrading bacteria isolated from Indonesian seawater. *Microbes Environ.* **22**, 412–415 (2007).

21. Wang, W. & Shao, Z. Diversity of flavin-binding monooxygenase genes (almA) in marine bacteria capable of degradation long-chain alkanes. *FEMS Microbiol. Ecol.* **80**, 523–533 (2012).

22. Lai, Q. *et al.* Parvibaculum indicum sp. nov., isolated from deep-sea water. *Int. J. Syst. Evol. Microbiol.* **61**, 271–274 (2011).

23. Harwati, T. U., Kasai, Y., Kodama, Y., Susilaningsih, D. & Watanabe, K. Tropicimonas isoalkanivorans gen. nov., sp. nov., a branched-alkane-degrading bacterium isolated from Semarang Port in Indonesia. *Int. J. Syst. Evol. Microbiol.* **59**, 388–391 (2009).

24. Harwati, T. U., Kasai, Y., Kodama, Y., Susilaningsih, D. & Watanabe, K. Tranquillimonas alkanivorans gen. nov., sp. nov., an alkane-degrading bacterium isolated from Semarang Port in Indonesia. *Int. J. Syst. Evol. Microbiol.* **58**, 2118–2121 (2008).

25. Davidova, I. A., Duncan, K. E., Choi, O. K. & Suflita, J. M. Desulfoglaeba alkanexedens gen. nov., sp. nov., an n-alkane-degrading, sulfate-reducing bacterium. *Int. J. Syst. Evol. Microbiol.* **56**, 2737–2742 (2006).

26. Kummer, C., Schumann, P. & Stackebrandt, E. Gordonia alkanivorans sp. nov., isolated from tar-contaminated soil. *Int. J. Syst. Evol. Microbiol.* **49**, 1513–1522 (1999).

27. Kim, S.-H. *et al.* Ketobacter alkanivorans gen. nov., sp. nov., an n-alkane-degrading bacterium isolated from seawater. *Int. J. Syst. Evol. Microbiol.* **68**, 2258–2264 (2018).

28. Liu, C. & Shao, Z. Alcanivorax dieselolei sp. nov., a novel alkane-degrading bacterium isolated from sea water and deep-sea sediment. *Int. J. Syst. Evol. Microbiol.* **55**, 1181–1186 (2005).

29. Wu, Y. *et al.* Alcanivorax hongdengensis sp. nov., an alkane-degrading bacterium isolated from surface seawater of the straits of Malacca and Singapore, producing a lipopeptide as its biosurfactant. *Int. J. Syst. Evol. Microbiol.* **59**, 1474–1479 (2009).

30. Yakimov, M. M. *et al.* Alcanivorax borkumensis gen. nov., sp. nov., a new, hydrocarbon-degrading and surfactant-producing marine bacterium. *Int. J. Syst. Evol. Microbiol.* **48**, 339–348 (1998).

31. Palleroni, N. J., Port, A. M., Chang, H.-K. & Zylstra, G. J. Hydrocarboniphaga effusa gen. nov., sp. nov., a novel member of the γ-Proteobacteria active in alkane and aromatic hydrocarbon degradation. *Int. J. Syst. Evol. Microbiol.* **54**, 1203–1207 (2004).

32. Schleheck, D., Tindall, B. J., Rossello-Mora, R. & Cook, A. M. Parvibaculum lavamentivorans gen. nov., sp. nov., a novel heterotroph that initiates catabolism of linear alkylbenzenesulfonate. *Int. J. Syst. Evol. Microbiol.* **54**, 1489–1497 (2004).

33. Harwati, T. U., Kasai, Y., Kodama, Y., Susilaningsih, D. & Watanabe, K. Tropicibacter naphthalenivorans gen. nov., sp. nov., a polycyclic aromatic hydrocarbon-degrading bacterium isolated from Semarang Port in Indonesia. *Int. J. Syst. Evol. Microbiol.* **59**, 392–396 (2009).

34. Rodrigo-Torres, L., Pujalte, M. J. & Arahal, D. R. Draft genome sequence of Thalassobius gelatinovorus CECT 4357T, a roseobacter with the potential ability to degrade polycyclic aromatic hydrocarbons. *Gene Reports* **9**, 32–36 (2017).

35. Gauthier, M. J. *et al.* Marinobacter hydrocarbonoclasticus gen. nov., sp. nov., a new, extremely halotolerant, hydrocarbon-degrading marine bacterium. *Int. J. Syst. Evol. Microbiol.* **42**, 568–576 (1992).

36. Peeters, S. H. *et al.* Description of Polystyrenella longa gen. nov., sp. nov., isolated from polystyrene particles incubated in the Baltic Sea. (2020).

37. Katayama, Y. *et al.* Effects of spilled oil on microbial communities in a tidal flat. *Mar. Pollut. Bull.* **47**, 85–90 (2003).

38. Wirth, J. S. & Whitman, W. B. Phylogenomic analyses of a clade within the roseobacter group suggest taxonomic reassignments of species of the genera Aestuariivita, Citreicella, Loktanella, Nautella, Pelagibaca, Ruegeria, Thalassobius, Thiobacimonas and Tropicibacter, and the proposal . *Int. J. Syst. Evol. Microbiol.* **68**, 2393–2411 (2018).

39. Pinto, M. *et al.* Putative degraders of low-density polyethylene-derived compounds are ubiquitous members of plastic-associated bacterial communities in the marine environment. *Environ. Microbiol.* **22**, 4779–4793 (2020).
